# Legume consumption in adults and risk of cardiovascular disease and type 2 diabetes: a systematic review and meta-analysis

**DOI:** 10.29219/fnr.v67.9541

**Published:** 2023-05-30

**Authors:** Birna Thorisdottir, Erik Kristoffer Arnesen, Linnea Bärebring, Jutta Dierkes, Christel Lamberg-Allardt, Alfons Ramel, Bright I. Nwaru, Fredrik Söderlund, Agneta Åkesson

**Affiliations:** 1 Faculty of Food Science and Nutrition, University of Iceland, Reykjavik, Iceland; 2 Department of Nutrition, Institute of Basic Medical Sciences, University of Oslo, Oslo, Norway; 3 Department of Internal Medicine and Clinical Nutrition, Institute of Medicine, Sahlgrenska Academy, University of Gothenburg, Gothenburg, Sweden; 4 Centre for Nutrition, Department of Clinical Medicine, University of Bergen, Bergen, Norway; 5 Mohn Nutrition Research Laboratory, Department of Clinical Science, University of Bergen, Bergen, Norway; 6 Department of Medical Biochemistry and Pharmacology, Haukeland University Hospital, Bergen, Norway; 7 Department of Food and Nutrition, University of Helsinki, Helsinki, Finland; 8 Krefting Research Centre, Institute of Medicine, University of Gothenburg, Gothenburg, Sweden; 9 Unit of Cardiovascular and Nutritional Epidemiology, Institute of Environmental Medicine, Karolinska Institutet, Stockholm, Sweden

**Keywords:** fabaceae, pulses, beans, lentils, Peas, human nutrition, cardiovascular diseases, diabetes mellitus type 2, non-communicable diseases

## Abstract

**Objectives:**

This study aimed to systematically review the evidence for associations between consumption of legumes and cardiovascular disease (CVD), type 2 diabetes (T2D) and their risk factors among healthy adults.

**Methods:**

We searched MEDLINE, Embase, Cochrane Central Register of Controlled Trials, and Scopus up to 16 May 2022 for ≥4 weeks long randomized (RCT) and non-randomized controlled trials and prospective cohort studies with follow-up ≥12 months, assessing legume intake (beans/lentils/peas/soybeans, excluding peanuts and legume-products/protein/powder/flour) as the intervention or exposure. Outcomes were CVD, coronary heart disease (CHD), stroke, T2D and in intervention trials only: changes in blood lipids, glycemic markers, and blood pressure. Risk of bias (RoB) was evaluated with Cochrane’s RoB2, ROBINS-I, and US Department of Agriculture (USDA)’s RoB-NObS. Effect sizes were pooled using random-effects meta-analyses and expressed as relative risk or weighed mean differences with 95% confidence intervals, heterogeneity quantified as *I^2^*. The evidence was appraised according to World Cancer Research Fund’s criteria.

**Results:**

Of the 181 full-text articles assessed for eligibility, 47 were included: 31 cohort studies (2,081,432 participants with generally low legume consumption), 14 crossover RCTs (448 participants), one parallel RCT and one non-randomized trial. Meta-analyses of cohort studies were suggestive of null associations for CVD, CHD, stroke and T2D. Meta-analyses of RCTs suggested a protective effect on total cholesterol (mean difference −0.22 mmol/L), low density lipoprotein (LDL)-cholesterol (−0.19 mmol/L), fasting glucose (−0.19 mmol/L), and HOMA-IR (−0.30). Heterogeneity was high (*I^2^* = 52% for LDL-cholesterol, >75% for others). The overall evidence for associations between consumption of legumes and risk of CVD and T2D was considered *limited – no conclusion*.

**Conclusion:**

Legume consumption was not found to influence risk of CVD and T2D in healthy adult populations with generally low legume consumption. However, protective effects on risk factors, seen in RCTs, lend some support for recommending legume consumption as part of diverse and healthy dietary patterns for prevention of CVD and T2D.

## Popular scientific summary

- Evidence from observational studies was suggestive of null associations between legume consumption and CVD and T2D.
- Meta-analyses of RCTs suggested a protective effect of legume consumption on established risk factors for CVD and T2D.
- Legume consumption may be recommended as a substitute for meat in sustainable diets and as part of a diverse and healthy dietary pattern for CVD and T2D prevention.

The global burden of disease study 2017 considered low legume intake a preventable risk factor for non-communicable diseases (1). The term ‘legumes’ includes various types of beans, lentils, peas, and soybeans. In addition, peanuts classify botanically as legumes, however in nutrition science they are classified as nuts. Legumes are excellent sources of essential amino acids, complex carbohydrates and fiber, are generally low in fat and saturated fatty acids, rich in micronutrients such as potassium, magnesium, folate, iron and zinc, as well as many bioactive compounds such as phytochemicals (2–5). From a sustainability perspective, legumes are considered important sources of dietary protein, as they have low greenhouse gas and water footprints, enrich the soil through nitrogen fixation, and reduce the need for fertilizers (6–8).

While legumes are used for human consumption worldwide, recent data indicate a universal lack of enough legumes in diets in most parts of the world, excluding some parts of Latin America, south Asia, and sub-Saharan Africa (1, 9). In Finland and Sweden, mean adult legume consumption is approximately 12 g/day and may be even lower in other Nordic and Baltic countries (10). The increasingly recognized need for the global population to shift towards a more sustainable, plant-based diet has driven an expansion in available research on health effects of legumes over the past years. This, along with the absence of specific recommendations on legume consumption in the 2012 edition of the Nordic Nutrition Recommendations (NNR); an emphasis in the NNR update for 2022 on healthy food groups that may act as substitutes for meat in sustainable diets; and an interest in possible associations between diets and cardiovascular disease (CVD) and type 2 diabetes (T2D) and associated biomarkers such as blood lipids, glycemic markers and blood pressure, all contributed to this being a prioritized topic for a systematic review in updating the NNR for 2022 (11).

Therefore, we performed a systematic review and meta-analysis of intervention trials and observational studies investigating the role of consumption of legumes in the development of CVD and T2D as well as their risk factors among generally healthy adults.

## Methods

This systematic review followed the guidelines developed for the 2022 revision of the NNR (12, 13) and preferred reporting for systematic reviews (14, 15). A study protocol was published prior to article selection in the PROSPERO database (https://www.crd.york.ac.uk, CRD42021250084). This systematic review is part of the NNR 2022 project, funded by the Nordic Council of Ministers and governmental food and health authorities of Norway, Finland, Sweden, Denmark, and Iceland (16).

### Eligibility criteria

The research question was developed by the NNR 2022 Committee and the NNR Systematic Review Centre (i.e. the authors) (Supplementary Table 1). The population of interest were adults in settings relevant for the general population in the Nordic and Baltic countries (i.e. free-living and generally healthy). Therefore, studies on patient groups (e.g. participants with established CVD or T2D at baseline) and weight-loss trials were excluded. The intervention/exposure of interest was legumes, excluding peanuts, and the comparator of interest were different intake levels in observational studies, and usual diet or other comparator in intervention studies. Legumes could be any type of fresh or cooked beans, lentils, peas, or soybeans consumed as a food. Specific legume types should be explored, if possible. Studies exploring processed legume products, such as legume protein/powder, legume flour, soy products (e.g. tofu), legume oils and legume snacks (with the exception of soynuts made from mature soybeans in intervention studies), were excluded, as were studies where the intervention/exposure of interest could not be separated from peanuts or vegetables. Substitution analyses (e.g. of replacing meat with legumes) were not included in this review. The outcomes of interest were incidence (non-fatal or fatal) of, or mortality from, CVD, coronary heart disease (CHD) and stroke, T2D incidence, and in intervention studies: changes in blood lipids, blood pressure, fasting glucose or insulin, and insulin resistance (homeostatic model assessment, HOMA-IR). Studies were included if they provided multivariable-adjusted risk estimates [risk ratios (RR), hazard ratios (HR), or odds ratios (OR)] or mean differences (intervention studies) with corresponding data to calculate the variance. Eligible randomized (RCT) and non-randomized controlled trials should have at least 4 weeks duration and prospective cohort studies should have at least 12 months of follow-up.

### Search strategy

A comprehensive literature search of MEDLINE, Embase, Cochrane Central Register of Controlled Trials and Scopus was performed at the Library of Medicine and Science at the University of Oslo, Norway up to the search date, initially on 2 May 2021, updated on 16 May 2022. The search strategy (Supplementary file 2) was developed in collaboration with the authors (led by BT and EKA) and peer-reviewed by research librarians at the Karolinska Institute in Stockholm, Sweden. Reference lists of relevant retrieved articles were also screened to identify additional articles. There were no publication date or language limitations in the search. Grey literature and unpublished study searches were not performed.

### Article selection and data collection

Pairs of two authors (CL-A, LB initial search, BT, AR updated search) were independently screened and selected studies for inclusion. The screening of titles/abstracts was performed in the web tool Rayyan (https://rayyan.qcri.org) in a blinded fashion. Discrepancies were resolved by discussion or by a third author (AÅ) both after title/abstract screening and full-text screening.

Data from full-text articles was extracted into pre-specified Excel extraction forms by pairs of two authors working independently (BIN, JD, FS initial search, BT, AR updated search). Among the data extracted were study design, information on participants and recruitment, exposure/interventions and controls, assessment of outcomes, follow-up, confounders, and results. When duplicate publications from the same study were identified, we included the report that included the largest number of cases for each endpoint of interest. In a few instances, study authors were contacted in an attemp to retrieve data considered necessary for meta-analyses.

### RoB assessment

Risk of bias of each included study was independently assessed as low, moderate/some concerns, serious or critical by two authors (EAR initial search, BT, AR updated search) using tools relevant for the different study types. Cochrane’s Risk of Bias 2 (RoB2) (17) was used for individually randomized, parallel-group trials, considering the RoB in five domains: arising from the randomization process, due to deviations from the intended interventions and missing outcome data, in the measurement of the outcome, and in selection of the reported result. A supplement to the main RoB2 (18) was used for crossover trials, additionally considering the RoB from period and carryover effects. Cochrane’s Risk of Bias in Non-randomized studies – of interventions (ROBINS-I) (19) was used for non-randomized intervention studies, and the ‘Risk of Bias for Nutrition Observational Studies’ (RoB-NObS) (20) developed by the USDA Nutrition Evidence Systematic Review (NESR) team was used for observational studies. Both ROBINS-I and RoB-NObS consider the RoB in seven domains, although with slightly different wording in signaling questions: due to confounding, in selection of participants, in classification of interventions/exposures, due to deviations from intended interventions/exposures, due to missing data, in measurement of outcomes, and in selection of the reported result. RoB was visualized by using the web app Risk-of-bias VISualization (robvis) (21).

### Synthesis methods

The evidence was synthesized qualitatively, based on study characteristics, context, strengths and limitations, heterogeneity, and relevance. Meta-analyses were performed if deemed appropriate to combine/pool the different studies, but only when more than three independent RCTs or five cohort studies with sufficient homogenous data existed (12). These conditions were met for total CVD and CVD mortality, total CHD and CHD incidence, total stroke and stoke incidence, T2D incidence, total cholesterol (TC), low density lipoprotein cholesterol (LDL-C), high density lipoprotein cholesterol (HDL-C), triglycerides (TG), fasting glucose, insulin, and HOMA-IR. All meta-analyses were performed using a random-effects model and the meta-analysis approach followed recommendations from the Agency for Healthcare Research and Quality (AHRQ) and the Cochrane’s Handbook (22, 23). In studies only reporting results stratified by sex or menopausal status, these estimates were first pooled within each study by an inverse variance fixed-effect model. Stratified analyses were performed if data allowed, by separating by incidence versus mortality in cohort studies, and further by legume type in both observational studies and RCTs. Sensitivity analyses were explored by overall RoB (RCTs alone).

For observational studies, the fully adjusted risk estimates [HR/RR; OR was converted into RR (24)] and 95% confidence intervals were log-transformed and summarized to assess the highest versus lowest consumption categories and dose-response relationships. For the dose-response analysis, additional required inputs for each category of exposure were the median exposure amount, person-years of follow-up, and number of cases. For estimating median exposure amounts in g/day in studies with an alternative reporting (such as servings/day or times/week), methods from Greenland 1987 were used (25). Consistent with most regional serving sizes (26) and in line with previous meta-analyses (27–29), we assumed that one serving of legumes equaled 100 g, if not otherwise specified. Dose-response effects/relationships were assessed by a one-stage approach estimating a pooled regression slope and standard error, from the dose-specific risk estimates within the studies included in the meta-analysis, thus indicating the association between a unit increase in legume intake and the respective outcomes with the lowest consumption category (usually 0 g/day) as reference (30). Potentially non-linear dose-response associations were explored by restricted cubic splines with knots fixed at the 10th, 50th and 90th percentiles of the exposure (31).

For RCTs, mean differences and their standard deviations (SDs) between the intervention and control group at the end of the intervention period were the primary effects of interest. For RCTs with more than one legume intervention arm, the results were first pooled into one intervention group with a fixed-effect meta-analysis, to avoid double-counting of participants (32). For crossover RCTs, we used results from paired analysis accounting for intra-individual correlation as reported, or calculated SD with a correlation coefficient of 0.6, which is considered a conservative estimate (33). Units were converted if needed: TC, LDL-C and HDL-C from mg/dL to mmol/L by dividing by 38.67, TG from mg/dL to mmol/L by dividing by 88.57, glucose from mg/dL to mmol/L by dividing by 18.02 and insulin from µIU/mL to pmol/L by multiplying by 6.00.

For all meta-analyses, potential heterogeneity between studies was assessed using the *I^2^* statistic. To assess small study effects, visual inspection of funnel plots and Egger’s regression test (significance level *P* > 0.1) were evaluated if there were at least 10 effect estimates (34). Meta-analyses were performed with the ‘meta’ command in Stata v17 (StataCorp LLC, College Station, Texas, USA).

### Strength of evidence assessment

Strength of evidence was appraised based on RoB, quantity, consistency, and precision in the evidence, according to the World Cancer Research Fund’s grading:

‘Convincing’, ‘Probable’, ‘Limited – suggestive, ‘Limited – no conclusion’, ‘Substantial effects unlikely’ (16). According to this classification, several conditions must be met for the body of evidence to be judged as *convincing*, that is, strong enough to support a causal relationship or *substantial effects unlikely*, that is strong enough to support that there is a convincing absence of a causal relationship. The evidence is considered as *probable* when it is strong enough to support that there is a probable causal relationship, with evidence from at least two independent cohort studies, no unexplained heterogeneity between- or within-study types, good-quality studies to confidentially exclude the possible random or systematic errors, and evidence for biological plausibility. The evidence is considered *limited – suggestive* when there is evidence suggestive of a consistent direction of effect, and evidence for biological plausibility. If the evidence is so limited that no firm conclusion can be made or there is inconsistency of direction of effect it is considered *limited – no conclusion*.

## Results

**Fig. 1:**
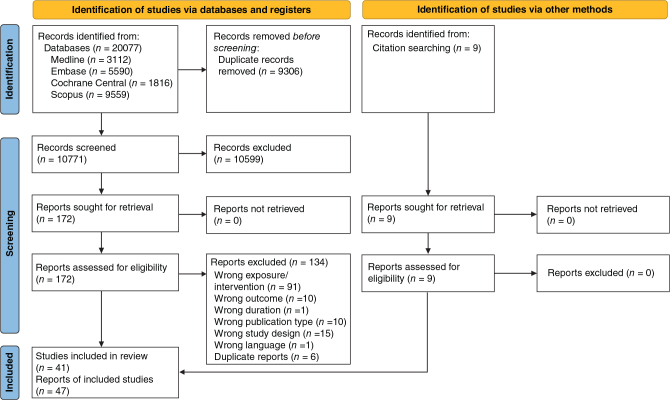
Flowchart of study selection. *From*: Page MJ, McKenzie JE, Bossuyt PM, Boutron I, Hoffmann TC, Mulrow CD, et al. The PRISMA 2020 statement: an updated guideline for reporting systematic reviews. BMJ 2021;372:n71. doi: 10.1136/bmj.n71. For more information, visit: http://www.prisma-statement.org/.

The database searches yielded 10,771 articles after deduplication, of which 10,599 were excluded after title/abstract screening. Nine additional records were identified by screening reference lists of retrieved articles and other systematic reviews. Of the 181 full-text articles evaluated, 47 met the criteria to be included in the review and 42 were included in quantitative synthesis (meta-analysis). Figure 1 gives the flowchart for the literature screening and Supplementary Table 2 lists the 134 articles excluded after full text screening.

### Overview of included studies

In total, 31 articles reporting possible observational associations between legume intake and cardiovascular endpoints (*n* = 21) or T2D (*n* = 10) and 16 articles reporting results from legume interventions on cardiometabolic risk factors were included (Table 1). They report results from 25 unique prospective cohorts, prospective observational analysis of participants from one RCT, as well as 13 unique crossover RCTs, one parallel RCT and one non-randomized trial.

**Table 1:** Characteristics of included studies.

Article, reference	Study design (cohort name, if applicable)	Country	Subjects	Age at entry	Number analyzed (% women)	Exposure	Follow-up (y) / Duration (weeks)	Outcomes	Funding	Risk of bias
***Observational studies, cardiovascular outcomes***										
Bernstein 2012 (89)	PC (NHS, HPFS)	USA	Female registered nurses; male health care professionals	30-55 (NSH), 40-75 (HPFS)	127160 (66)	Legumes	26 (NHS), 22 (HPFS)	Stroke	Public	Moderate
Blekkenhorst 2017 (90)	PC (PLSAW)	Australia	Elderly women	≥70	1226 (100)	Legumes	15	CVD	Public	Moderate
Farvid 2017 (91)	PC (GCS)	Iran	General population	36-85	42403 (57)	Legumes	11	CVD, CHD, Stroke	Mixed	Moderate
Fraser 1992 (92)	PC (AHS)	USA	Seventh-day Adventists	≥25	26743 (63)	Legumes	6	CHD	Public	Serious
Fung 2018 (93)	PC (NHS, NHS II, HPFS)	USA	Female registered nurses; male health care professionals	30-55 (NHS), 25-42 (NSH II), 40-75 (HPFS)	212142 (79)	Legumes	28 (NHS), 20 (NHS II), 24 (HPFS)	CHD	Public	Moderate
Golzarand 2022 (51)	PC (TLGS)	Iran	General population	20-69	2863 (56)	Legumes	11	CVD	Not reported	Moderate
Haring 2014 (94)	PC (ARIC)	USA	General population	45-65	12066 (56)	Legumes	22	CHD	Public	Moderate
Haring 2015 (95)	PC (ARIC)	USA	General population	45-65	11601 (56)	Legumes	22	Stroke	Public	Moderate
Im & Park 2021 (41)	PC (Ansan-Ansung)	South Korea	General population, urban and rural	40-69	4713 (100)	Soybeans	7	CVD	Public	Moderate
Kokubo 2007 (37)	PC (JPHC)	Japan	General population	40-59	40462 (52)	Soybeans	13	CVD, CHD, Stroke	Public	Moderate
Martinez-Gonzales 2011 (96)	PC (SUN)	Spain	University graduates from all Spain	>25	14129 (60)	Legumes	5	CVD, CHD	Public	Moderate
Miller 2017 (45)	PC (PURE)	18 countries in seven geographical regions1	General population	35-70	135335 (58)	Legumes	7	CVD, CHD, Stroke	Mixed	Moderate
Mizrahi 2009 (35)	PC (FMC)	Finland	General population	40-74	3932 (48)	Legumes	24	Stroke	Public	Moderate
Nagura 2009 (38)	PC (JACC)	Japan	General population	40-79	59485 (58)	Soybeans	13	CVD, CHD, Stroke	Public	Moderate
Nouri 2021 (52)	PC (ICS)	Iran	General population	≥35	5432 (51)	Legumes	13	CVD	None	Serious
Papandreou 2019 (53)	prospective analysis from PREDIMED	Spain	Adults at high risk of CVD	55-80	7212 (57)	Legumes	6	CVD	Mixed	Moderate
Perez-Cornago 2021 (44)	PC (EPIC)	10 European countries2	General population	35-65	490311 (71)	Legumes	13	CHD	Mixed	Moderate
Stefler 2017 (97)	PC (HAPIEE)	Poland, Russia and Czech Republic	General population	45-69	19263 (55)	Legumes	7	CVD, CHD, Stroke	Public	Moderate
Tong 2020 (43)	PC (EPIC)	9 European countries3	General population	35-65	418329 (67)	Legumes	13	Stroke	Public	Moderate
Yamasaki 2015 (39)	PC (JMS)	Japan	General population	mostly 40-69	11066 (61)	Soybeans	12	CVD	Private	Serious
Yu 2014 (98)	PC (SWHS, SMHS)	China	Urban population	40-70 (SWHS), 40-74 (SMHS)	122685 (55)	Legumes	10 (SWHS), 5 (SMHS)	CHD	Public	Moderate
***Observational studies, diabetes***										
Bazzano 2008 (57)	PC (NHS)	USA	Female registered nurses	38-63	71346 (100)	Legumes	18	T2D	Public	Moderate
Becerra-Tomás 2018 (55)	prospective analysis from PREDIMED	Spain	Adults at high risk of CVD	55-80	3349 (62)	Legumes	4	T2D	Mixed	Moderate
Ericson 2013 (36)	PC (MDC)	Sweden	General population	45-74	27140 (61)	Legumes	12	T2D	Mixed	Moderate
Hodge 2004 (46)	PC (MCCS)	Australia	Australia-born and migrants from Southern Europe	40-69	36787 (unclear)	Legumes	4	T2D	Public	Moderate
Khalili-Moghadam 2018 (54)	PC (TLGS)	Iran	General population	20-69	2139 (55)	Legumes	6	T2D	Not reported	Serious
Liu 2004 (99)	PC (WHS)	USA	Female health professionals	≥45	38018 (100)	Legumes	9	T2D	Not reported	Moderate
Meyer 2000 (100)	PC (IWHS)	USA	Postmenopausal women	55-69	35988 (100)	Legumes	6	T2D	Public	Serious
O’Connor 2020 (56)	PC (ARIC)	USA	General population	45-65	11991 (56)	Legumes	22	T2D	Public	Moderate
Villegas 2008 (42)	PC (SWHS)	China	Female urban population	40-70	64191 (100)	Legumes and soybeans	5	T2D	Public	Moderate
Yan 2021 (40)	PC (JACC)	Japan	General population	40-79	21925 (62)	Soybeans	5	T2D	Public	Moderate
***Intervention studies***										
Abeysekara 2012 (58)	crossover trial	Canada	Healthy adults over 50 y, no hypolipidemic medications (n=20 had high cholesterol)	≥50	80 (approx. 71)	Legumes	8	risk factors	Mixed	Some concerns
Azadbakht 2007 (59)	crossover trial	Iran	Postmenopausal women with MetS	postmenopausal women	42 (100)	Soy-nut	8	risk factors	Public	Some concerns
Bakhtiari 2019 (60)	parallel trial	Iran	Postmenopausal women with MetS	60-70	25 (100)	Soy-nut	12	risk factors	Public	Low
Cobiac 1990 (101)	crossover trial	Australia	Mildly hypercholesterolemic men, healthy and no hypolipidemic medications	29-65	20 (0)	Beans	4	risk factors	Private	Some concerns
Doma 2021 (61)	crossover trial	Canada	Adults with elevated LDL-cholesterol, no hypolipidemic medications	≥18 Y (mean±SD: 48±14)	66 (approx. 56)	Beans	4	risk factors	Mixed	Low
Duane 1997 (65)	crossover trial	USA	Healthy men living on metabolic ward for the purpose of the study	41-78	9 (0)	Legumes	6	risk factors	Private	Some concerns
Lin 1981 (62)	non-randomized trial	China	Adults with asymptomatic hyperlipidemia	27-66	Invervention n = 73, Control n = 169 (unclear)	Beans	12	risk factors	Not reported	Critical
Mackay 1992 (47)	crossover trial	New Zealand	Hypercholesterolaemic adults	28-66	39 (44)	Beans	6	risk factors	Mixed	Some concerns
Mizelman 2020 (67)	crossover trial	Canada	Healthy young athletes	young (mean±SD 22±6)	10 (50)	Legumes	4	risk factors	Private	Some concerns
Nestel 2004 (48)	crossover trial	Australia	Healthy adults, no hypolipidemic medications	30-70	20 (50)	Chickpeas	6	risk factors	Private	Some concerns
Pittaway 2006 (49)	crossover trial	Australia	Healthy adults, no hypolipidemic medications	30-70	47 (60)	Chickpeas	5-6	risk factors	Private	Some concerns
Pittaway 2007 (50)	crossover trial	Australia	Healthy adults, no hypolipidemic medications	30-70	27 (63)	Chickpeas	5	risk factors	Private	Some concerns
Saraf-Bank 2016 (68)	crossover trial	Iran	Healthy first-degree relatives of patients with diabetes	50±7	26 (54)	Legumes	6	risk factors	Public	Some concerns
Tischmann 2022 (66)	crossover trial	the Netherlands	Healthy older adults	60-70	23 (52)	Soy-nut	16	risk factors	Private	Low
Winham 2007a (64)	crossover trial	USA	Healthy adults, fasting insulin level 15-50 µU/ml, no cholesterol-lowering medications	22-65	16 (56)	Pinto beans, black eyed peas	8	risk factors	Mixed	Some concerns
Winham 2007b (63)	crossover trial	USA	Hypercholesterolaemic adults	22-70	23 (57)	Beans	8	risk factors	Private	Some concerns

Abbreviations: PC, prospective cohort; FFQ, food frequency questionnaire; FR, food record.

Abbreviated cohort names: AHS, Adventist Health Study; ARIC, Atherosclerosis Risk in Communities, EPIC, European Prospective Investigation into Cancer and Nutrition; FMC, Finnish Mobile Clinic Health Examination Survey; GCS, Golestan Cohort Study; HAPIEE, Health Alcohol and Psychosocial Factors in Eastern Europé; HPFS, Health Professionals Follow-Up Study; ICS, Isfahan cohort study; IWHS, Iowa Women’s Health Study; JACC, Japan Collaborative Cohort Study; JMS, Jichi Medical School Cohort Study; JPHC, Japan Public Health Center-based Prospective Study; MCCS, Melbourne Collaborative Cohort Study; MDC, Malmö Diet and Cancer Study; NHS, Nurses Health Study; PLSAW, Perth Longitudinal Study of Aging in Women; PREDIMED, Prevención con Dieta Mediterránea; PURE, Prospective Urban Rural Epidemiology; SUN, Seguimiento Universidad de Navarra Study; SMHS, Shanghai Men’s Health Study; SWHS, Shanghai Women’s Health Study; TLGS, Tehran Lipid and Glucose Study; WHS, Women’s health study.

1Miller 2017, 18 countries: Canada, Sweden, United Arab Emirates, Argentina, Brazil, Chile, Poland, Turkey, Malaysia, South Africa, China, Colombia, Iran, the occupied Palestinian territory, Bangladesh, India, Pakistan, Zimbabwe.

2Perez-Cornago 2021, 10 European countries: Denmark, France, Germany, Greece, Italy, The Netherlands, Norway, Spain, Sweden, and the UK.

3Tong 2020, 9 European countries: Denmark, Germany, Greece, Italy, the Netherlands, Norway, Spain, Sweden, and the UK.

### Characteristics of observational studies

Summarizing from Table 1 and Supplementary Table 3, the observational studies analyzed 2,081,432 participants (69% women) from Europe, USA, Australia, China, Iran, Japan, and South Korea, and one study combined data from 18 countries in seven geographical regions. Except for the PREDIMED cohort, the participants were obtained from generally healthy populations. One US cohort included a large proportion of vegetarian participants, but results were only presented for the overall population (38). The mean age was 53 years at baseline, ranging in individual studies from 38 to 75 years. Follow-up time ranged from 4 to 28 years in individual studies (mean 12 years) and person-years ranged from 6,986 to 6,170,299 in the studies for which they were reported. Most observational studies (*n* = 20) were publicly funded.

Diets were assessed by food frequency questionnaire in all but the Nordic studies, which used a diet history (47, 58). More than half of the studies (*n* = 17) assessed diet only at baseline, while others had two (*n* = 6) or more (*n* = 8) diet assessments. The exposure assessed was most often total legumes (beans, lentils, peas, occasionally soybeans), while studies from Japan, South Korea and one study from China only assessed intake of soybeans (43, 44, 48, 54, 64, 65). By a crude approximation we estimated the median intake in the studies to be 20 g/day, but it varied between studies and geographical regions. The Finnish study and the European EPIC study reported medians below 10 g/day, while the PURE study including 18 countries worldwide and studies from South Korea and Australia reported medians of 40–50 g/day (43, 46, 47, 51, 53, 59).

Outcomes assessed were CVD (5 studies reporting incidence, 7 studies reporting mortality), CHD (8 studies reporting incidence, 3 studies reporting mortality), stroke (6 studies reporting incidence, 3 studies reporting mortality), and T2D (10 studies). In total, 51,454 cardiovascular events and 15,992 diabetes events occurred. The CVD endpoints were most often assessed by reviewing medical records and/or death certificates. In some studies, especially those on T2D, participants or their families self-reported the outcome. Most often measures were taken to check the reported outcomes using official records or other sources. Most studies adjusted for important confounders including age, sex, body mass index, smoking, alcohol use, physical activity, energy intake, relevant medication use or history/family history of relevant diseases or conditions.

### Characteristics of intervention studies

Summarizing from Table 1 and Supplementary Table 4, the crossover RCTs analyzed 448 participants (58% women) from Australia, New Zealand, USA, Canada, Iran, and the Netherlands, the parallel-group RCT analyzed 25 participants from Iran and the non-randomized trial analyzed 242 participants from China, although the number reporting was unclear in that article. Most trials included generally healthy participants, some with hypercholesterolemia or metabolic syndrome (MetS), but did not include participants using lipid-lowering medications. The age range was wide (≤30 to ≥65 years) in 10 studies, one studied young athletes, and the remaining five studied middle aged or older adults. The duration of the crossover RCTs ranged from 4 to 16 weeks (mean 7 weeks), while the parallel-group and non-randomized trials had a duration of 12 weeks. Blood lipids were assessed in 14 studies, glucose or insulin or HOMA-IR in 11 studies and blood pressure in 2 studies. Most intervention studies (*n* = 8) were industry funded.

The interventions were consumption of either a mixture of legumes (*n* = 4) or individual types; beans (*n* = 5), soybeans (*n* = 3), chickpeas (*n* = 3) and individual effects of pinto beans and black-eyed peas (*n* = 1). Various methods were used to assess adherence to the intervention. Exposure amounts were in the ranges of 37–156 g/day (dry weight) for mixed legumes, 50–377 g/day (cooked weight) for beans, 30–67 g/day for soynuts and 130–140 g/day (cooked weight) for chickpeas, pinto beans, and black-eyed peas. A few of the included studies were considered not having, for the purpose of the present systematic review, an optimal control intervention; Mackay compared beans with low or high fibre oat bran (73) while Nestel and Pittaway compared chickpeas with a whole grain wheat diet (75–77).

### Risk of bias in included studies

**Fig. 2:**
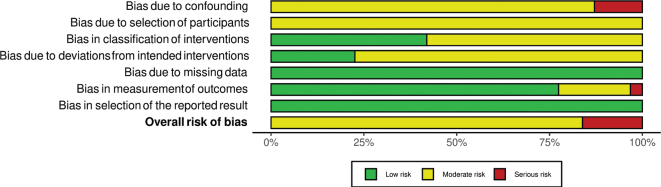
Summary risk of bias per domain in cohort studies (*n* = 31).

**Fig. 3:**
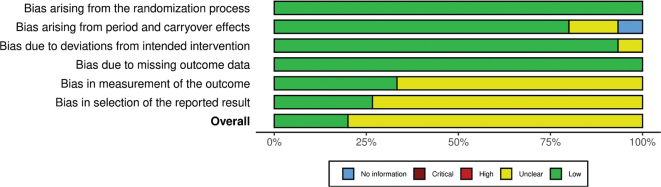
Summary risk of bias per domain in RCTs (*n* = 15).

The summary RoB assessments of the included cohort studies and RCTs are shown in Figs. 2 and 3, respectively. Study level RoB assessments are shown in Supplementary Fig. 1 (cohort studies and the single non-randomized trial) and Supplementary Fig. 2 (RCTs, both crossover and parallel).

The overall RoB in cohort studies was regarded as moderate for 27 articles and serious for 4 articles. While some potential for confounding of the effect of exposure was expected in all studies, those not measuring energy intake (identified as a key confounder) were regarded as having serious RoB in domain 1. Some bias in domain 2 was expected for all studies since the start of follow-up was long after participants likely started consuming legumes. When outcome assessment was based on hospital registries, death certificates, or otherwise confirmed, the RoB for measurement of outcomes (domain 6) was regarded as low, while self-reported assessments that were partly confirmed were regarded as moderate RoB, and when possibly invalid, as serious RoB.

The overall RoB in the non-randomized intervention was regarded as critical due to issues with confounding and missing data. The RoB for three RCTs was regarded as low and moderate for the remaining 12 RCTs. The lack of a washout period raised concerns (domain 1), as did low compliance (domain 2), lack of blinding of outcome assessors (domain 4) and lack of a published analysis plan, especially considering the private funding (domain 5).

### Summary of findings from observational studies

Five individual observational studies (four regarded with moderate and one with serious RoB) reported significant associations between legume intake and CVD outcomes in fully adjusted models (see Supplementary Table 3). Four reported inverse associations: two between total legume intake and CVD incidence (40, 49), one between total legume intake and ischemic stroke (but not total stroke) (47) and one between soybean intake and CVD mortality (48). On the other hand, a prospective analysis from the PREDIMED trial reported an association between total legume intake and higher CVD mortality (50).

**Fig. 4:**
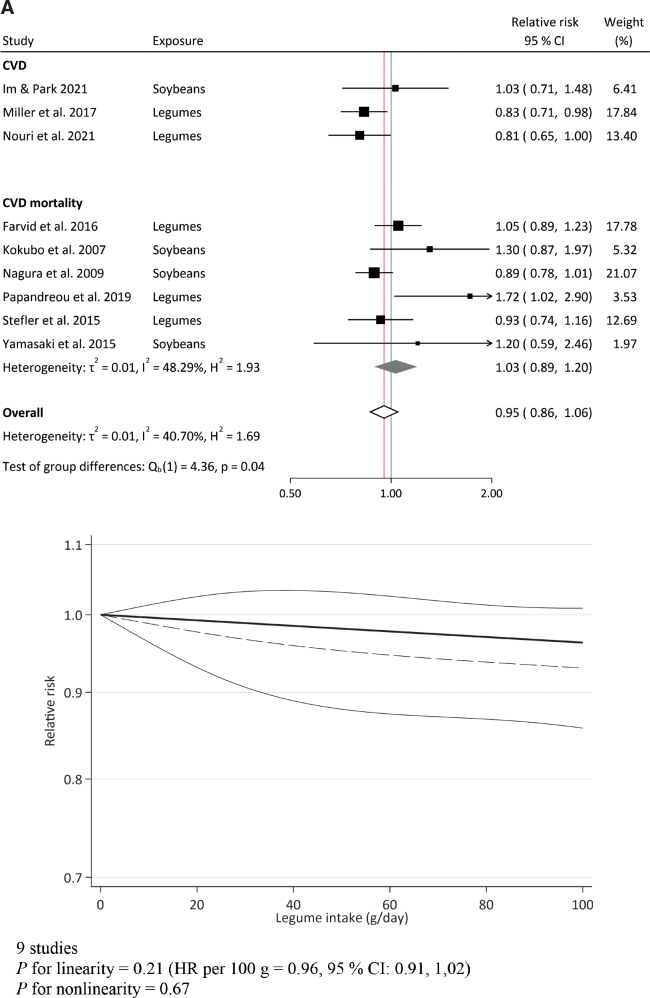

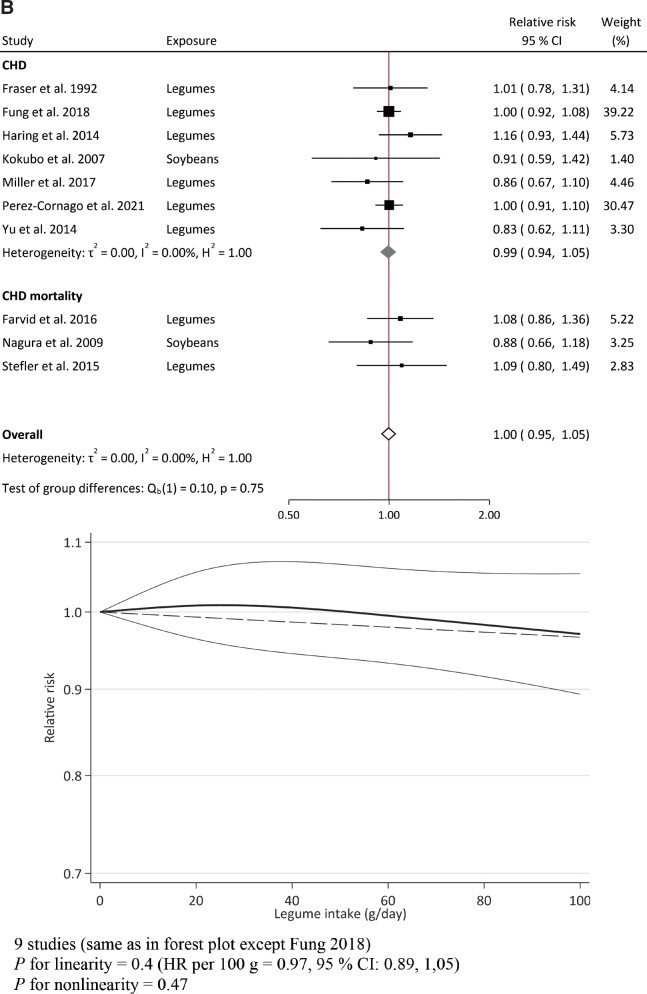

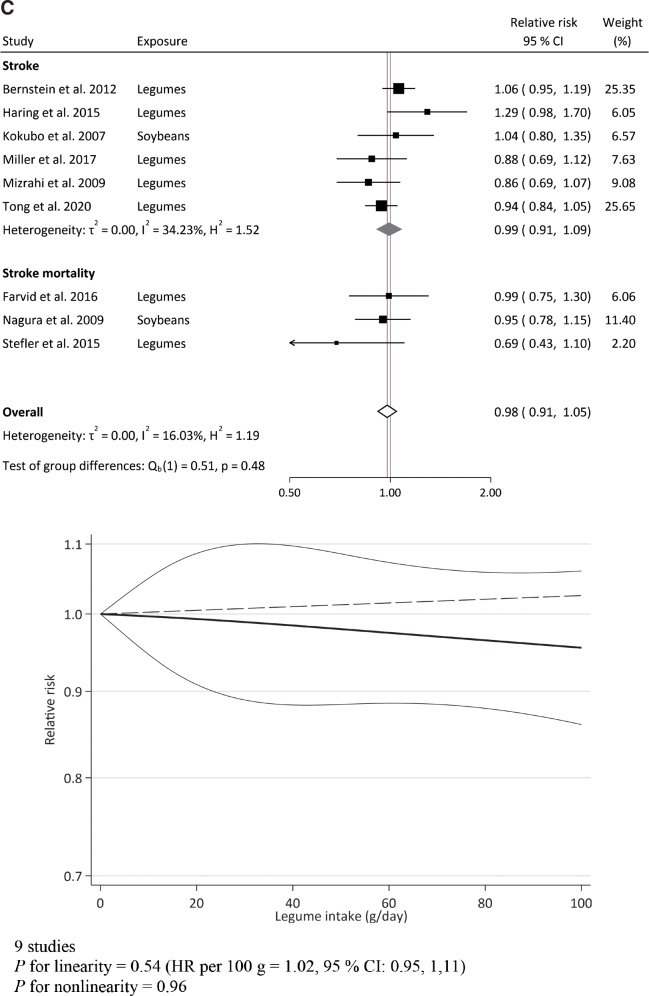

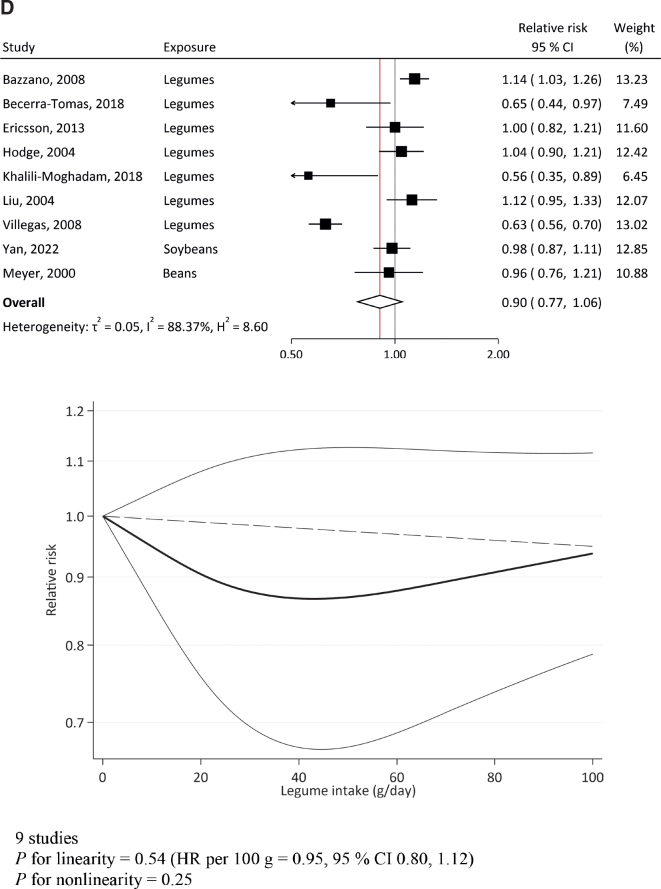
Legume consumption and risk of cardiometabolic endpoints in cohort studies, shown overall and separated by incidence and mortality: CVD (A), CHD (B), stroke (C), T2D (D). The upper figures show summary forest plots of pooled relative risk estimates between highest and lowest legume consumption categories and risk of the endpoints. The lower figures show linear (dashed line) and non-linear (solid line) dose-response associations (with 95% CI) between legume consumption and risk of the endpoints (total CVD, total CHD, total stroke, T2D), with 0 g/day as reference and vertical axes log transformed.

Out of the 12 studies reporting on overall CVD, nine provided sufficient data to be included in the meta-analysis (3 studies on incidence and 6 studies on mortality). The analysis resulted in a pooled estimate for high versus low legume intake of RR 0.95 (95% CI, 0.86, 1.06, *I^2^* = 41%) for total (or ‘overall’) CVD. An assessment of CVD mortality alone resulted in a pooled estimate of RR 1.03 (95% CI, 0.89, 1.20, *I^2^* = 48%) **(**Fig. 4A). For CHD, 10 out of 11 studies provided sufficient data to be included in the high versus low legume intake meta-analysis (7 studies on incidence and 3 on mortality). The analysis resulted in a pooled estimate of RR 1.00 (95% CI, 0.95, 1.05, *I^2^* = 0%) for total (‘overall’) CHD and an estimate of RR 0.99 (95% CI, 0.94, 1.05, *I^2^* = 0%) for CHD incidence alone (Fig. 4B). For stroke, all nine studies retrieved (6 on incidence and 3 on mortality) were included in the high versus low meta-analysis; RR 0.98 (95% CI, 0.91, 1.05, *I^2^* = 16%) for total (‘overall’) stroke and RR 0.99 (95% CI, 0.91, 1.09, *I^2^* = 34%) for stroke incidence alone (Fig. 4C). No clear dose-response association was found for any of the outcomes.

Half of the studies on legume intake and T2D risk (four regarded with moderate and one with serious RoB) reported significant associations. Becerra-Tomás, Khalili-Moghadam, O’Connor and Villegas reported inverse associations between total legume intake and T2D (57, 60, 63, 64). Furthermore, lentils and soybeans were inversely associated with T2D risk in Becerra-Tomás and Villegas (64), respectively. In the opposite direction, Bazzano reported significant associations between legume intake and higher T2D (56). Nine out of 10 studies reporting on T2D provided sufficient data to be included in the meta-analysis, resulting in a pooled estimate for legume intake of RR 0.90 (95% CI, 0.77, 1.06, *I^2^* = 88%) and no clear dose response association (Fig. 4D).

Separate assessment by legume type (overall legumes excluding soybeans vs. soybeans alone) resulted in pooled estimates for high versus low legume (excluding soybean) intake of RR 1.00 (95% CI, 0.95, 1.06, *I^2^* = 0%) for overall CHD, RR 0.97 (95% CI, 0.89, 1.07, *I^2^* = 35%) for overall stroke and RR 0.89 (95% CI, 0.74, 1.07, *I^2^* = 89%) for T2D (Supplementary Fig. 3). There were no differences between studies based on funding source (not shown).

### Summary of findings from intervention studies

As shown in Supplementary Table 4, 12 out of the 16 intervention studies reported some significant effects, all considered protective for the outcomes of interest. A significant lowering of TC and/or LDL-C was observed in 10 intervention studies (65–68, 70–72, 76, 79–81); the effect on TC was observed in 8 studies and LDL-C also in 8 studies. These studies included healthy middle-aged or older adults, some with high cholesterol, and postmenopausal women with MetS, in interventions on mixed legumes (≥120–150 g/day dry weight), soynut (≥30 g/day), beans (≥50 g/day cooked weight), chickpeas (140 g/day cooked weight) or pinto beans (130 g/day cooked weight). Significant increases in HDL-cholesterol was observed in studies on hypercholesterolemic adults consuming beans (80 g/day cooked weight) and healthy young athletes consuming mixed legumes (156 g/day dry weight) (73, 74). Changes in insulin, glucose and HOMA-IR were only seen in RCTs from Iran on postmenopausal women with MetS consuming 30–35 g/day soynuts (67, 68). No effects were seen on blood pressure (67, 78).

**Fig. 5:**
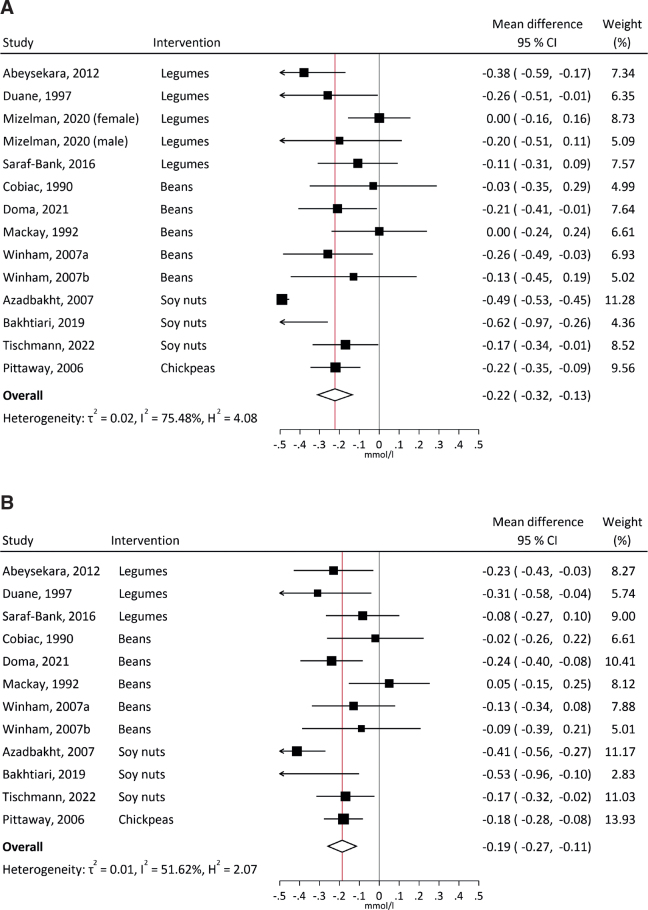

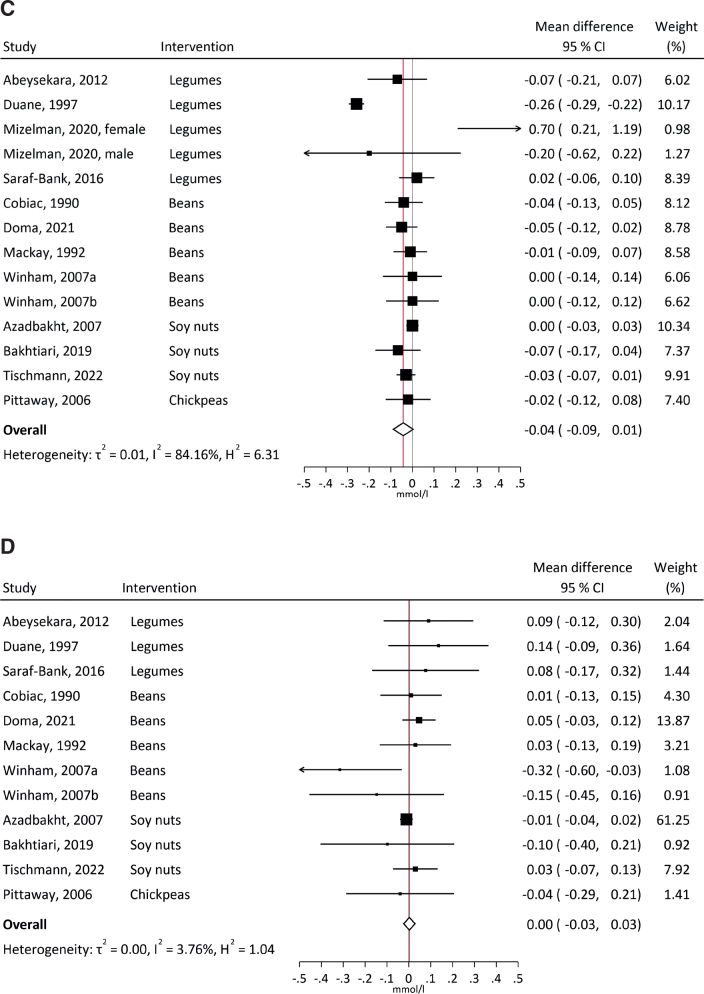

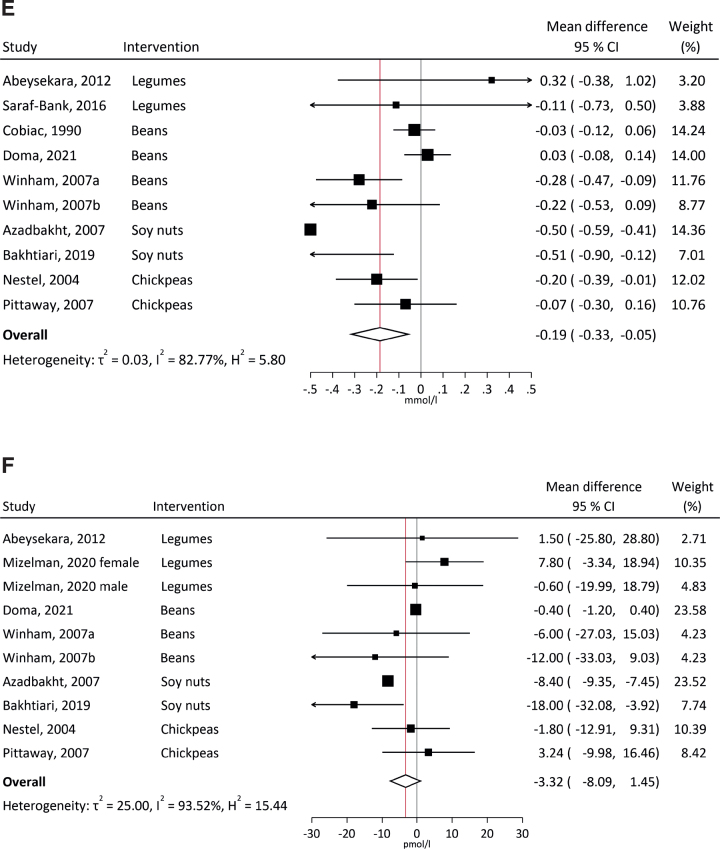

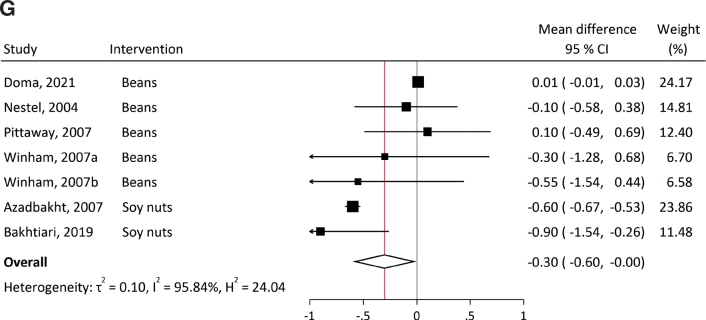
Effect of legume intervention versus control in RCTs. The figures show summary forest plots of pooled mean differences between intervention and control in cardiometabolic risk factors: total cholesterol, mmol/L (A), LDL-cholesterol, mmol/L (B), HDL-cholesterol, mmol/L (C), triglycerides, mmol/L (D), glucose, mmol/L (E), insulin, pmol/L (F), HOMA-IR (G).

All RCTs (crossover and parallel) were included in the meta-analysis, while the non-randomized trial was not (72). The summary effect sizes (Fig. 5) showed significantly decreased TC (-0.22 mmol/L, 95% CI, -0.32, -0.13, *I^2^* = 75%), LDL-cholesterol (-0.19 mmol/L, 95% CI, -0.27, -0.11, *I^2^* = 52%), fasting glucose (-0.19 mmol/L, 95% CI, -0.33, -0.05, *I^2^* = 83%), and HOMA-IR (-0.30, 95% CI, -0.60, -0.00, *I^2^* = 96%) with legume interventions compared with controls, but insignificant effects on HDL-C, TG, and insulin. Heterogeneity was high but decreased when separated by type of legumes (Supplementary Fig. 4). Especially, excluding the soynut trials removed nearly all heterogeneity. Excluding soynut trials, the intervention effect of legumes was -0.17 mmol/L (95% CI -0.25, -0.09, *I^2^* = 31%) for TC, -0.15 mmol/L (95% CI -0.21, -0.09, *I^2^* = 5%) for LDL-C, -0.09 mmol/L (95% CI -0.19, 0.01, *I^2^* = 50%) for glucose and 0.01 (95% CI -0.01, 0.03, *I^2^* = 0%) for HOMA-IR. The effects of soynuts on TC, fasting glucose and insulin were significantly different from other interventions (*P* = 0.03 for TC, *P* = 0.003 for glucose and *P* < 0.001 for insulin). For glucose, the intervention effect was also larger in studies 6 weeks or longer than 4–5 weeks (data not shown). Subgroup analysis by RoB gave similar results for RCTs with some concerns and all studies (Supplementary Fig. 5). The effect sizes did not vary by funding source (not shown), except for larger effects in the two publicly funded RCTs using soynuts for glucose, insulin, and HOMA-IR.

### Reporting bias

Based on inspection of funnel plots (Supplementary Fig. 6), and Egger’s test (in meta-analyses with 10 or more comparators), we did not find evidence of publication bias in the form of small study-effects bias. Egger’s test for legumes and CHD gave a *P*-value of 0.62, and for the risk factors the *P*-values ranged from 0.38 to 0.98, with the exception of HDL-C (*P* = 0.06) where one small trial found a large increase in females (*n* = 5).

### Certainty in the evidence

Overall, the findings from this systematic review are mixed, with high heterogeneity and mostly moderate/some concern for RoB (Table 2). Although it was not the purpose of this review, we consider the evidence strong in supporting the absences of any adverse effects of legume intake in relation to CVD or T2D. Nevertheless, meta-analyses of prospective cohort studies on either total CVD, CHD, stroke or T2D showed null associations comparing higher with lower legume consumption and no dose–response relationship. This would indicate substantial effects unlikely but needs to be interpreted in the light of the generally low legume consumption reported in the cohorts. RCTs on approximately >120–150 g legumes per day suggested a biological plausibility, with most, but not all studies reporting lowering of TC and LDL-C and meta-analyses additionally suggesting protective effects on glucose and HOMA-IR.

**Table 2:** Summary of findings

Outcome	Study type	Studies total (n included in meta-analysis)	Exposure (n studies)	Association/effect (n studies)	Meta-analysis results	Heterogeneity (I2)	Risk of bias (n studies)	Evidence assessment
CVD	Observational	12 (9)	Legumes (8) Soybeans (4)	↑ 1 ↓ 3 ↔ 8	0.95 (0.86, 1.06) 1 No clear dose-response	40.70%	Moderate: 10 Serious: 2	Mixed findings, in favor of null association
CHD	Observational	11 (10)	Legumes (9) Soybeans (2)	↑ 0 ↓ 0 ↔ 11	1.00 (0.95, 1.05) 1 No clear dose-response	0.00%	Moderate: 10 Serious: 1	In favor of null association
Stroke	Observational	9 (9)	Legumes (7) Soybeans (2)	↑ 0 ↓ 1 ↔ 8	0.98 (0.91, 1.05) 1 No clear dose-response	16.03%	Moderate: 9	In favor of null association
T2D	Observational	10 (9)	Legumes (9) Soybeans (1)	↑ 1 ↓ 4 ↔ 5	0.90 (0.77, 1.06) 1 No clear dose-response	88.37%	Moderate: 8 Serious: 2	Mixed findings, in favor of null association
Total cholesterol	Interventions	14 (13)	Mixed (4) Beans (5) Soy-nuts (3) Chickpeas (1) Pinto/black eyed (1)	↑ 0 ↓ 8 ↔ 6	-0.22 (-0.32, -0.13) mmol/L 2	75.48%	Low: 3 Some concern: 10 Critical: 1	Suggestions of a protective effect
LDL-cholesterol	Interventions	12 (12)	Mixed (3) Beans (4) Soy-nuts (3) Chickpeas (1) Pinto/black eyed (1)	↑ 0 ↓ 8 ↔ 4	-0.19 (-0.27, -0.11) mmol/L 2	51.62%	Low: 3 Some concern: 9	Suggestions of a protective effect
HDL- cholesterol	Interventions	13 (13)	Mixed (4) Beans (4) Soy-nuts (3) Chickpeas (1) Pinto/black eyed (1)	↑ 2 ↓ 0 ↔ 11	-0.04 (-0.09, 0.01) mmol/L 2	84.16%	Low: 3 Some concern: 10	In favor of no effect
Triglycerides	Interventions	13 (12)	Mixed (3) Beans (5) Soy-nuts (3) Chickpeas (1) Pinto/black eyed (1)	↑ 0 ↓ 0 ↔ 13	0.00 (-0.03, 0.03) mmol/L 2	3.76%	Low: 3 Some concern: 9 Critical: 1	In favor of no effect
Glucose	Interventions	10 (10)	Mixed (2) Beans (3) Soy-nuts (2) Chickpeas (2) Pinto/black eyed (1)	↑ 0 ↓ 1 ↔ 9	-0.19 (-0.33, -0.05) mmol/L 2	82.77%	Low: 2 Some concern: 8	Mixed findings, some suggestions of a protective effect
Insulin	Interventions	9 (9)	Mixed (2) Beans (2) Soy-nuts (2) Chickpeas (2) Pinto/black eyed (1)	↑ 0 ↓ 1 ↔ 8	-3.32 (-8.09, 1.45) pmol/L 2	93.52%	Low: 2 Some concern: 7	Mixed findings, no conclusion
HOMA-IR	Interventions	7 (7)	Mixed (0) Beans (2) Soy-nuts (2) Chickpeas (2) Pinto/black eyed (1)	↑ 0 ↓ 2 ↔ 5	-0.30 (-0.60, -0.00) 2	95.84%	Low: 2 Some concern: 7	Mixed findings, some suggestions of a protective effect
Systolic blood pressure	Interventions	2 (0)	Mixed (1) Soy-nuts (1)	↑ 0 ↓ 0 ↔ 2	-	-	Some concern (2)	No conclusion
Diastolic blood pressure	Interventions	2 (0)	Mixed (1) Soy-nuts (1)	↑ 0 ↓ 0 ↔ 2	-	-	Some concern (2)	No conclusion

Abbreviations: CHD, coronary heart disease; CVD, cardiovascular disease; HOMA-IR, homeostatic model assessment for insulin resistance; T2D, type 2 diabetes;

1RR (95% CI), comparing high vs. low consumption

2Mean difference (95% CI), comparing intervention vs. control

Since legume interventions were suggested to have protective effects on blood lipids and glycemic markers, we did not consider the evidence strong enough to support a convincing absence of a causal relationship. However, the direction of effect in observational studies was not considered consistent enough to be suggestive of an association (as in the grading *limited – suggestive*). Therefore, we judged the evidence for associations between legume intake and CVD or T2D as *limited – no conclusion*.

## Discussion

This comprehensive systematic review and meta-analysis of both observational and intervention studies is, to our knowledge, the most recent update on the evidence for associations between legume intake and CVD and T2D and their risk factors. While legume RCTs were found having favorable effects on blood lipids and glycemic markers, which are risk factors for CVD and T2D (1), this systematic review found little support, but also little opposition, for recommending dietary inclusion of legumes for the purpose of CVD and T2D prevention for healthy general populations in the Nordic and Baltic countries. Data were too limited to draw conclusions about blood pressure or specific types of legumes. The evidence for associations between legume intake and CVD or T2D was considered *limited – no conclusion*.

### Comparison with other reviews and discussion on heterogeneity

Previous systematic reviews and meta-analyses on legumes have similarly found conflicting evidence, often considered of low or limited strength. Some, but not all systematic reviews on observational studies have found evidence for an association of overall legume intake with decreased risk of CVD and/or CHD in high versus low comparison of included studies or dose-response analysis (28, 29, 82–85). We are not aware of systematic reviews reporting evidence for an association between legume intake and stroke (28, 29, 82, 86, 87). Previous systematic reviews have also assessed the evidence for specific types of legumes, notably finding no or some evidence for soy intake and CVD (88–90). Weak or null associations between legume intake and T2D have been found (91) and recently, intakes of tofu, soy protein, and soy isoflavones, but not total legumes or total soy, were found to be associated with lower T2D incidence (92). This is interesting in the light of our observed differences between effects of soynut and other legume interventions in crossover trials. Meta-analyses on RCTs have previously found suggestions of protective effects of diets rich in legumes on TC and LDL-cholesterol (93, 94). Another recent systematic review without meta-analyses on RCTs of legume interventions found evidence for improvements in blood lipid profile, blood pressure, inflammation biomarkers, and other measures resulting from consumption of approximately 150 g/day of legumes, similar to our finding (95), while only significant effects of interventions on glycemic control were found for individuals with established T2D in another review of RCTs including individuals with or without T2D or prediabetes (96).

The different findings in the current and some previous systematic reviews and meta-analyses may depend on many factors. Combining incidence and mortality outcomes, combining consumption of soy and other legumes, and pooling findings for men and women when reported separately may, for example, potentially result in heterogeneity in findings where null findings for some may mask findings for others. For example, in a meta-analysis from 2017, heterogeneity was considered to be due to null results of stroke, affecting estimates for total CVD (82). In our analyses, excluding soynut trials removed nearly all statistical heterogeneity in intervention studies, while separating by type of legumes (overall legumes vs. soy) did not affect heterogeneity in observational studies.

The setting, amounts of legumes consumed and broader dietary context of legume consumption are other important factors to consider and need further investigation. A recent meta-analysis found legume intake to be associated with higher T2D in Europe, mainly driven by studies from Germany, UK, and Sweden; but there is no evidence of associations between legume intake and T2D in the Americas, Eastern Mediterranean, and Western Pacific (91). Interestingly, in our analyses on legume consumption and T2D, the studies with relative risks >1.10 were from the USA, while the studies with RR ≤0.65 were from Iran, China and Spain (a population consuming a Mediterranean-style diet). Residual confounding may be one source of this difference, for example, different overall dietary patterns and cooking methods of legumes (91), confounding by other components in the diet [e.g. consumption of legumes as parts of meat-based meals in Western Europe (97) and possibly the USA versus consumption of legumes with olive oil in the Mediterranean region (98)].

### Strengths and limitations

The main strength of this review is the established process for undertaking robust systematic reviews. A priori, the NNR 2022 Committee established criteria for the prioritization and selection of systematic review topics. Prior to undertaking the review, we developed and registered a detailed protocol to enhance transparency in the review process (12, 13). We searched four leading electronic databases to identify relevant studies on the review topic, these databases cover most of the literature in medicine and public health. The search was updated before publication, to be as up to date as possible. In addition to this comprehensive database search, we screened reference lists from included articles and relevant systematic reviews for additional potentially eligible studies. We included nine articles from this additional search, which may seem high, but we believe that it can all be explained by the exposure characterization, as legumes were often explored as a subgroup of vegetables, thus not mentioned in title/abstract. After our thorough article searching, we consider it unlikely that we missed any relevant literature, although it cannot be excluded. The review processes were rigorously implemented, with independent assessments taken at each stage, including literature screening and data extraction.

All observational studies were classified as having either moderate or serious RoB. The RCTs were classified as having low or some concerns for RoB, with the exception for the non-randomized trial. The RoB assessment tool we used for cohort studies is more appropriate for evaluating causality than commonly used summary score-based quality appraisal tools, and since it partly assesses whether a cohort study mimics a RCT, it is very difficult for a study to get a low RoB. That being said, most of the included cohort studies were prone to some of the limitations inherent in many observational epidemiologic studies, including risk of residual confounding and risk associated with selection of participants into the study and drop-outs. We found in general no significant differences between studies with different sources of funding. However, it should be recognized that almost all RCTs had private or mixed private/public funding.

Since another systematic review by the same authors examined the evidence for whether replacing animal protein with plant protein reduces risk of CVD and T2D, substitution effects were not considered in the current review. Therefore, the comparison in observational studies was done on different exposure amounts, which sometimes needed to be estimated and are in general sensitive to risk of misclassification of exposure status and possibilities of change in exposure status. Findings from RCTs were mostly based on consumption of >120 g/day of legumes or specific types of legumes, which is very high considering the low consumption seen in the Nordic countries (9, 10).

### Conclusion and public health relevance

Overall, the findings from this systematic review and meta-analysis were mixed, and evidence from observational studies, generally with low legume consumption, was suggestive of null associations. However, protective effects seen in RCTs on established risk factors for CVD and T2D lend some support for recommending legume consumption as part of a diverse and healthy dietary pattern for CVD and T2D prevention in healthy general populations in the Nordic and Baltic countries. Even though the effect sizes seemed clinically small, they may still be relevant in a life-course perspective on the population level (99). It should be noticed that the amounts of legumes were commonly higher in RCTs (>120–150 g/day legumes) than the mean in the highest intake category in cohort studies. Although the current intake in some Western countries may possibly be higher than what was commonly consumed in the cohort studies included in this review, we believe that intervention trials exploring effects of lower consumption levels on cardiometabolic biomarkers would be of public health relevance. Although the evidence for associations between legume intake and CVD or T2D was graded as *limited – no conclusion*, we consider the current results will inform the updated recommendations in the 2022 edition of NNR, which was the primary purpose of the review.

## Registration

PROSPERO registration number CRD42021250084.
