# Supplementary material for: Legume consumption in adults and risk of cardiovascular disease and type 2 diabetes: a systematic review and meta-analysis
Source: Food Nutr Res. 2023 May 30;67:10.29219/fnr.v67.9541. doi: 10.29219/fnr.v67.9541 (PMC10243120; doi:10.29219/fnr.v67.9541)
Supplement: Supplementary file 1 [file FNR-67-9541-s001.docx]

**Supplementary table 1.** Details of the research question as specified in the pre-registered protocol.

| **Population** | **Intervention or Exposure** | **Comparators** | **Outcomes** | **Timing** | **Setting** | **Study design** |
| --- | --- | --- | --- | --- | --- | --- |
| Adults 18 years and older | Consumption of pulses/legumes (subgroups if possible), excluding peanuts | In intervention studies, the intervention should be compared to usual diet or other comparator.  In cohort studies: No/low vs. high consumption (e.g. in quartiles or quintiles).  Dose- response. | Atherosclerotic cardiovascular disease including:  Major incident fatal and non-fatal CVD (combined or separate: myocardial infarction, stroke, coronary heart disease, coronary artery bypass graft  CVD mortality  Incident T2D  Changes in insulin resistance, insulin sensitivity, HBA1c, fasting glucose and insulin  Changes in blood pressure, blood lipids | 12 months for prospective studies, 1 month for clinical trials | Relevant for the general population in the Nordic and Baltic countries | Randomized or non-randomized intervention trials.  For observational epidemiological studies, we will consider prospective cohort studies, nested case-control studies, and case-cohort studies. |

Footnotes:

Abbreviations: CVD, cardiovascular disease; T2D, type 2 diabetes

**Supplementary table 2.** Excluded articles after full-text screening, along with reasons.

| **Article** | **Reason** |
| --- | --- |
| Bakhtiary, A., et al., *Effects of soy on metabolic biomarkers of cardiovascular disease in elderly women with metabolic syndrome.* Archives of Iranian Medicine, 2012. **15**(8): p. 462-468. | duplicate reports (participants were included in another publication (Bakhtiary 2019)) |
| Bakhtiary, A., et al., *Evaluation of the oxidative stress and glycemic control status in response to soy in older women with the metabolic syndrome.* Iranian Red Crescent Medical Journal, 2011. **13**(11): p. 795-804. | duplicate reports (participants were included in another publication (Bakhtiary 2019)) |
| Joshipura, K.J., et al., *Fruit and vegetable intake in relation to risk of ischemic stroke.* Jama, 1999. **282**(13): p. 1233-1239. | duplicate reports (participants were included in another publication (Bernstein 2012)) |
| Liu, W., et al. *Fruit, vegetable, and legume intake and the risk of all-cause, cardiovascular, and cancer mortality: A prospective study.* Clinical nutrition. 2021. **40**(6), p.4316-4323. | duplicate reports (population included in another article (Miller 2017)) |
| Misirli, G., et al., *Relation of the traditional mediterranean diet to cerebrovascular disease in a mediterranean population.* American Journal of Epidemiology, 2012. **176**(12): p. 1185-1192. | duplicate reports (participants were included in another publication (Tong 2020)) |
| Nouri, F., et al., *Intake of legumes and the risk of cardiovascular disease: frailty modeling of a prospective cohort study in the Iranian middle-aged and older population.* European Journal of Clinical Nutrition, 2016. **70**(2): p. 217-221. | duplicate reports (participants were included in another publication (Nouri 2021)) |
| Grande, F., J.T. Anderson, and A. Keys, *Effect of Carbohydrates of Leguminous Seeds, Wheat and Potatoes on Serum Cholesterol Concentration in Man.* Journal of Nutrition, 1965. **86**: p. 313-317. | wrong duration |
| Abiemo, E.E., et al., *Relationships of the Mediterranean dietary pattern with insulin resistance and diabetes incidence in the Multi-Ethnic Study of Atherosclerosis (MESA).* British Journal of Nutrition, 2013. **109**(8): p. 1490-1497. | wrong exposure/intervention |
| Acharjee, S., et al., *Effect of soy nuts and equol status on blood pressure, lipids and inflammation in postmenopausal women stratified by metabolic syndrome status.* Metabolism: Clinical & Experimental, 2015. **64**(2): p. 236-243. | wrong exposure/intervention (legume protein/powder) |
| Ashton, E. and M. Ball, *Effects of soy as tofu vs meat on lipoprotein concentrations.* European Journal of Clinical Nutrition, 2000. **54**(1): p. 14-19. | wrong exposure/intervention |
| Ashton, E.L., F.S. Dalais, and M.J. Ball, *Effect of meat replacement by tofu on CHD risk factors including copper induced LDL oxidation.* Journal of the American College of Nutrition, 2000. **19**(6): p. 761-767. | wrong exposure/intervention |
| Back, H.I., et al., *Effects of Chungkookjang supplementation on obesity and atherosclerotic indices in overweight/obese subjects: a 12-week, randomized, double-blind, placebo-controlled clinical trial.* Journal of Medicinal Food, 2011. **14**(5): p. 532-537. | wrong exposure/intervention |
| Bazzano, L.A., et al., *Legume consumption and risk of coronary heart disease in US men and women: NHANES I Epidemiologic Follow-up Study.* Archives of Internal Medicine, 2001. **161**(21): p. 2573-2578. | wrong exposure/intervention (peanuts and peanut butter included as legumes) |
| Byun, M.S., et al., *Korean traditional Chungkookjang improves body composition, lipid profiles and atherogenic indices in overweight/obese subjects: a double-blind, randomized, crossover, placebo-controlled clinical trial.* European Journal of Clinical Nutrition, 2016. **70**(10): p. 1116-1122. | wrong exposure/intervention |
| Chiechi, L.M., et al., *The effects of a soy rich diet on serum lipids: the Menfis randomized trial.* Maturitas, 2002. **41**(2): p. 97-104. | wrong exposure/intervention |
| Cryne, C.N., et al., *Spray-dried pulse consumption does not affect cardiovascular disease risk or glycemic control in healthy males.* Food Research International, 2012. **48**(1): p. 131-139. | wrong exposure/intervention (legume protein/powder) |
| Ding, M., et al., *Consumption of soy foods and isoflavones and risk of type 2 diabetes: A pooled analysis of three US cohorts.* European Journal of Clinical Nutrition, 2016. **70**(12): p. 1381-1387. | wrong exposure/intervention |
| Escobedo et al. Common Bean Baked Snack Consumption Reduces Apolipoprotein B-100 Levels: A Randomized Crossover Trial | wrong exposure/intervention (snack made from beans) |
| Feskens, E.J., et al., *Dietary factors determining diabetes and impaired glucose tolerance. A 20-year follow-up of the Finnish and Dutch cohorts of the Seven Countries Study.* Diabetes Care, 1995. **18**(8): p. 1104-1112. | wrong exposure/intervention |
| Fruhbeck, G., I. Monreal, and S. Santidrian, *Hormonal implications of the hypocholesterolemic effect of intake of field beans (Vicia faba L.) by young men with hypercholesterolemia.* American Journal of Clinical Nutrition, 1997. **66**(6): p. 1452-1460. | wrong exposure/intervention (legume flour) |
| Fung, T.T., et al., *Dietary patterns and the risk of coronary heart disease in women.* Archives of Internal Medicine, 2001. **161**(15): p. 1857-1862. | wrong exposure/intervention |
| Fung, T.T., et al., *Dietary patterns, meat intake, and the risk of type 2 diabetes in women.* Archives of Internal Medicine, 2004. **164**(20): p. 2235-2240. | wrong exposure/intervention |
| Fung, T.T., et al., *Prospective study of major dietary patterns and stroke risk in women.* Stroke, 2004. **35**(9): p. 2014-2019. | wrong exposure/intervention |
| Gardner-Thorpe, D., et al., *Dietary supplements of soya flour lower serum testosterone concentrations and improve markers of oxidative stress in men.* European Journal of Clinical Nutrition, 2003. **57**(1): p. 100-106. | wrong exposure/intervention (legume flour) |
| Ghafarzadeh, M. and M. Namdari, *Effect of soybean diet on serum lipids an lipoproteins of postmenopausal women.* Pakistan Journal of Medical Sciences, 2010. **26**(2): p. 407-410. | wrong exposure/intervention (legume protein/powder) |
| Goldin, B.R., et al., *Hormonal response to diets high in soy or animal protein without and with isoflavones in moderately hypercholesterolemic subjects.* Nutrition & Cancer, 2005. **51**(1): p. 1-6. | wrong exposure/intervention |
| Gonciulea, A.R. and D.E. Sellmeyer, *The effect of dietary protein source on serum lipids: Secondary data analysis from a randomized clinical trial.* Journal of Clinical Lipidology, 2017. **11**(1): p. 46-54. | wrong exposure/intervention |
| Hall, R.S., et al., *Lupin kernel fibre-enriched foods beneficially modify serum lipids in men.* European Journal of Clinical Nutrition, 2005. **59**(3): p. 325-333. | wrong exposure/intervention (legume flour) |
| Ham, J.O., et al., *Endocrinological response to soy protein and fiber in mildly hypercholesterolemic men.* Nutrition Research, 1993. **13**(8): p. 873-884. | wrong exposure/intervention |
| Haub, M.D., A.M. Wells, and W.W. Campbell, *Beef and soy-based food supplements differentially affect serum lipoprotein-lipid profiles because of changes in carbohydrate intake and novel nutrient intake ratios in older men who resistive-train.* Metabolism: Clinical & Experimental, 2005. **54**(6): p. 769-774. | wrong exposure/intervention |
| Heidemann, C., et al., *A dietary pattern protective against type 2 diabetes in the European Prospective Investigation into Cancer and Nutrition (EPIC)--Potsdam Study cohort.* Diabetologia, 2005. **48**(6): p. 1126-1134. | wrong exposure/intervention |
| Heidemann, C., et al., *Dietary patterns and risk of mortality from cardiovascular disease, cancer, and all causes in a prospective cohort of women.* Circulation, 2008. **118**(3): p. 230-237. | wrong exposure/intervention |
| Hodge, A.M., et al., *Dietary patterns and diabetes incidence in the Melbourne Collaborative Cohort Study.* American Journal of Epidemiology, 2007. **165**(6): p. 603-610. | wrong exposure/intervention |
| Hodgson, J.M., et al., *Effects of increasing dietary protein and fibre intake with lupin on body weight and composition and blood lipids in overweight men and women.* International Journal of Obesity, 2010. **34**(6): p. 1086-1094. | wrong exposure/intervention (legume flour) |
| Hoevenaar-Blom, M.P., et al., *Mediterranean style diet and 12-year incidence of cardiovascular diseases: the EPIC-NL cohort study.* PLoS ONE [Electronic Resource], 2012. **7**(9): p. e45458. | wrong exposure/intervention |
| Hoscan, Y., F. Yigit, and H. Muderrisoglu, *Adherence to Mediterranean diet and its relation with cardiovascular diseases in Turkish population.* International journal of clinical and experimental medicine, 2015. **8**(2): p. 2860-2866. | wrong exposure/intervention |
| Hosseinpour-Niazi, S., et al., *Cereal, fruit and vegetable fibre intake and the risk of the metabolic syndrome: a prospective study in the Tehran Lipid and Glucose Study.* Journal of Human Nutrition & Dietetics, 2015. **28**(3): p. 236-245. | wrong exposure/intervention |
| Jenkins, D.J., et al., *Combined effect of vegetable protein (soy) and soluble fiber added to a standard cholesterol-lowering diet.* Metabolism: Clinical & Experimental, 1999. **48**(6): p. 809-816. | wrong exposure/intervention |
| Jenkins, D.J., et al., *Effects of high- and low-isoflavone soyfoods on blood lipids, oxidized LDL, homocysteine, and blood pressure in hyperlipidemic men and women.* American Journal of Clinical Nutrition, 2002. **76**(2): p. 365-372. | wrong exposure/intervention (legume protein/powder) |
| Jung, S.M., et al., *A Non-Probiotic Fermented Soy Product Reduces Total and LDL Cholesterol: A Randomized Controlled Crossover Trial.* Nutrients, 2021. **13**(2): p. 06. | wrong exposure/intervention |
| Katagiri, R., et al., *Association of soy and fermented soy product intake with total and cause specific mortality: Prospective cohort study.* The BMJ, 2020. **368**. | wrong exposure/intervention |
| Kim, H., et al., *Plant-based diets and incident metabolic syndrome: Results from a South Korean prospective cohort study.* PLoS Medicine / Public Library of Science, 2020. **17**(11): p. e1003371. | wrong exposure/intervention |
| Kim, Y. and Y. Je, *A modified Mediterranean diet score is inversely associated with metabolic syndrome in Korean adults.* European Journal of Clinical Nutrition, 2018. **72**(12): p. 1682-1689. | wrong exposure/intervention |
| Koloverou, E., et al., *Adherence to Mediterranean diet and 10-year incidence (2002-2012) of diabetes: correlations with inflammatory and oxidative stress biomarkers in the ATTICA cohort study.* Diabetes/Metabolism Research Reviews, 2016. **32**(1): p. 73-81. | wrong exposure/intervention |
| Koloverou, E., et al., *Dietary Patterns and 10-year (2002-2012) Incidence of Type 2 Diabetes: Results from the ATTICA Cohort Study.* The Review of Diabetic Studies, 2016. **13**(4): p. 246-256. | wrong exposure/intervention |
| Konishi, K., et al., *Dietary Soy Intake Is Inversely Associated with Risk of Type 2 Diabetes in Japanese Women but Not in Men.* Journal of Nutrition, 2019. **149**(7): p. 1208-1214. | wrong exposure/intervention |
| Kurowska, E.M., et al., *Effects of substituting dietary soybean protein and oil for milk protein and fat in subjects with hypercholesterolemia.* Clinical & Investigative Medicine - Medecine Clinique et Experimentale, 1997. **20**(3): p. 162-170. | wrong exposure/intervention |
| Lee, K.W., et al., *Identification of Dietary Patterns Associated with Incidence of Hyperglycemia in Middle-Aged and Older Korean Adults.* Nutrients, 2019. **11**(8): p. 04. | wrong exposure/intervention |
| Lee, Y.P., et al., *Effects of lupin kernel flour-enriched bread on blood pressure: a controlled intervention study.* American Journal of Clinical Nutrition, 2009. **89**(3): p. 766-772. | wrong exposure/intervention (legume flour) |
| Liu, Z.M., et al., *Whole soy, but not purified daidzein, had a favorable effect on improvement of cardiovascular risks: a 6-month randomized, double-blind, and placebo-controlled trial in equol-producing postmenopausal women.* Molecular Nutrition & Food Research, 2014. **58**(4): p. 709-717. | wrong exposure/intervention (legume protein/powder) |
| Ma, L., et al., *Isoflavone Intake and the Risk of Coronary Heart Disease in US Men and Women: Results From 3 Prospective Cohort Studies.* Circulation, 2020. **141**(14): p. 1127-1137. | wrong exposure/intervention (tofu) |
| Mitrou, P.N., et al., *Mediterranean dietary pattern and prediction of all-cause mortality in a US population: Results from the NIH-AARP diet and health study.* Archives of Internal Medicine, 2007. **167**(22): p. 2461-2468. | wrong exposure/intervention |
| Mollard, R.C., et al., *Regular consumption of pulses for 8 weeks reduces metabolic syndrome risk factors in overweight and obese adults.* British Journal of Nutrition, 2012. **108**: p. S111-122. | wrong exposure/intervention |
| Morimoto, A., et al., *Effects of healthy dietary pattern and other lifestyle factors on incidence of diabetes in a rural Japanese population.* Asia Pacific Journal of Clinical Nutrition, 2012. **21**(4): p. 601-608. | wrong exposure/intervention |
| Morimoto, Y., et al., *Soy consumption is not protective against diabetes in Hawaii: the Multiethnic Cohort.* European Journal of Clinical Nutrition, 2011. **65**(2): p. 279-282. | wrong exposure/intervention |
| Moslehi, N., et al., *Patterns of food consumption and risk of type 2 diabetes in an Iranian population: A nested case-control study.* Nutrition and Dietetics, 2016. **73**(2): p. 169-176. | wrong exposure/intervention |
| Mueller, N.T., et al., *Soy intake and risk of type 2 diabetes in Chinese Singaporeans corrected.* European Journal of Nutrition, 2012. **51**(8): p. 1033-1040. | wrong exposure/intervention |
| Murkies, A.L., et al., *Dietary flour supplementation decreases post-menopausal hot flushes: Effect of soy and wheat.* Maturitas, 1995. **21**(3): p. 189-195. | wrong exposure/intervention (soy flour) |
| Nadia, F.S., et al., *The effect of processed tempeh gembus to triglycerides levels and insulin resistance status in women with obesity.* Food Research, 2020. **4**(4): p. 1000-1010. | wrong exposure/intervention |
| Nagata, C., et al., *Dietary soy and natto intake and cardiovascular disease mortality in Japanese adults: the Takayama study.* American Journal of Clinical Nutrition, 2017. **105**(2): p. 426-431. | wrong exposure/intervention |
| Nanri, A., et al., *Soy product and isoflavone intakes are associated with a lower risk of type 2 diabetes in overweight Japanese women.* Journal of Nutrition, 2010. **140**(3): p. 580-586. | wrong exposure/intervention |
| Nettleton, J.A., et al., *Dietary patterns and risk of incident type 2 diabetes in the Multi-Ethnic Study of Atherosclerosis (MESA).* Diabetes Care, 2008. **31**(9): p. 1777-1782. | wrong exposure/intervention |
| Nguyen, H.N., et al., *Dietary tofu intake and long-term risk of death from stroke in a general population.* Clinical Nutrition, 2018. **37**(1): p. 182-188. | wrong exposure/intervention (tofu) |
| Nozue et al. Fermented soy products intake and risk of cardiovascular disease and total cancer incidence: The Japan Public Health Center-based Prospective study | wrong exposure/intervention (soy products) |
| Nozue, M., et al., *Fermented Soy Product Intake Is Inversely Associated with the Development of High Blood Pressure: The Japan Public Health Center-Based Prospective Study.* Journal of Nutrition, 2017. **147**(9): p. 1749-1756. | wrong exposure/intervention |
| Nozue, M., et al., *Fermented soy products intake and risk of cardiovascular disease and total cancer incidence: The Japan Public Health Center-based Prospective study.* European Journal of Clinical Nutrition, 2020. **4**: p. 04. | wrong exposure/intervention |
| Odegaard, A.O., et al., *Dietary patterns and incident type 2 diabetes in chinese men and women: the singapore chinese health study.* Diabetes Care, 2011. **34**(4): p. 880-885. | wrong exposure/intervention |
| Okuda, N., et al., *Fruit and vegetable intake and mortality from cardiovascular disease in Japan: a 24-year follow-up of the NIPPON DATA80 Study.* European Journal of Clinical Nutrition, 2015. **69**(4): p. 482-488. | wrong exposure/intervention |
| Oosthuizen, W., et al., *Extruded dry beans and serum lipoprotein and plasma haemostatic factors in hyperlipidaemic men.* European Journal of Clinical Nutrition, 2000. **54**(5): p. 373-379. | wrong exposure/intervention (legume flour) |
| Padhi, E.M., et al., *Whole Soy Flour Incorporated into a Muffin and Consumed at 2 Doses of Soy Protein Does Not Lower LDL Cholesterol in a Randomized, Double-Blind Controlled Trial of Hypercholesterolemic Adults.* Journal of Nutrition, 2015. **145**(12): p. 2665-2674. | wrong exposure/intervention (legume flour) |
| Potter, S.M., et al., *Depression of plasma cholesterol in men by consumption of baked products containing soy protein.* American Journal of Clinical Nutrition, 1993. **58**(4): p. 501-506. | wrong exposure/intervention |
| Roughead, Z.K., et al., *Controlled substitution of soy protein for meat protein: effects on calcium retention, bone, and cardiovascular health indices in postmenopausal women.* Journal of Clinical Endocrinology & Metabolism, 2005. **90**(1): p. 181-189. | wrong exposure/intervention |
| Ruscica, M., et al., *Effect of soy on metabolic syndrome and cardiovascular risk factors: a randomized controlled trial.* European Journal of Nutrition, 2018. **57**(2): p. 499-511. | wrong exposure/intervention (soy products) |
| Santiago-Torres, M., et al., *Development and Use of a Traditional Mexican Diet Score in Relation to Systemic Inflammation and Insulin Resistance among Women of Mexican Descent.* Journal of Nutrition, 2015. **145**(12): p. 2732-2740. | wrong exposure/intervention |
| Sapbamrer, R., N. Visavarungroj, and M. Suttajit, *Effects of dietary traditional fermented soybean on reproductive hormones, lipids, and glucose among postmenopausal women in northern Thailand.* Asia Pacific Journal of Clinical Nutrition, 2013. **22**(2): p. 222-228. | wrong exposure/intervention |
| Satija, A., et al., *Plant-Based Dietary Patterns and Incidence of Type 2 Diabetes in US Men and Women: Results from Three Prospective Cohort Studies.* PLoS Medicine / Public Library of Science, 2016. **13**(6): p. e1002039. | wrong exposure/intervention |
| Shi, Z. and V. Ganji, *Dietary patterns and cardiovascular disease risk among Chinese adults: a prospective cohort study.* European Journal of Clinical Nutrition, 2020. **74**(12): p. 1725-1735. | wrong exposure/intervention |
| Shi, Z., et al., *Dietary patterns associated with hypertension risk among adults in Thailand: 8-year findings from the Thai Cohort Study.* Public Health Nutrition, 2019. **22**(2): p. 307-313. | wrong exposure/intervention |
| Shimazu, T., et al., *Dietary patterns and cardiovascular disease mortality in Japan: a prospective cohort study.* International Journal of Epidemiology, 2007. **36**(3): p. 600-609. | wrong exposure/intervention |
| Shin, S.K., et al., *Supplementation of Cheonggukjang and red ginseng Cheonggukjang can improve plasma lipid profile and fasting blood glucose concentration in subjects with impaired fasting glucose.* Journal of Medicinal Food, 2011. **14**(1): p. 108-113. | wrong exposure/intervention |
| Simao, A.N., et al., *Blood pressure decrease with ingestion of a soya product (kinako) or fish oil in women with the metabolic syndrome: role of adiponectin and nitric oxide.* British Journal of Nutrition, 2012. **108**(8): p. 1435-1442. | wrong exposure/intervention (legume flour) |
| Simao, A.N., M.A. Lozovoy, and I. Dichi, *Effect of soy product kinako and fish oil on serum lipids and glucose metabolism in women with metabolic syndrome.* Nutrition, 2014. **30**(1): p. 112-115. | wrong exposure/intervention (legume flour) |
| Strengers, J.G., et al., *The association of the Mediterranean diet with heart failure risk in a Dutch population.* Nutrition, Metabolism and Cardiovascular Diseases, 2021. **31**(1): p. 60-66. | wrong exposure/intervention |
| Talaei, M., et al., *Dietary soy intake is not associated with risk of cardiovascular disease mortality in Singapore Chinese adults.* Journal of Nutrition, 2014. **144**(6): p. 921-928. | wrong exposure/intervention (soy protein) |
| Tektonidis, T.G., et al., *A Mediterranean diet and risk of myocardial infarction, heart failure and stroke: A population-based cohort study.* Atherosclerosis, 2015. **243**(1): p. 93-98. | wrong exposure/intervention |
| Thirunavukkarasu, D., N.H. Kirubamani, and M.B. Naidu, *The effect of soy flour intake on systemic blood pressure and glycemic control in post-menopausal women with pre-diabetes and prehypertension.* Indian Journal of Pharmaceutical Education and Research, 2017. **51**(2): p. 349-354. | wrong exposure/intervention (legume flour) |
| Voortman, T., et al., *Adherence to the 2015 Dutch dietary guidelines and risk of non-communicable diseases and mortality in the Rotterdam Study.* European Journal of Epidemiology, 2017. **32**(11): p. 993-1005. | wrong exposure/intervention |
| Wang et al. Consumption of soy products and cardiovascular mortality in people with and without cardiovascular disease: a prospective cohort study of 0.5 million individuals | wrong exposure/intervention (no separate analysis for soybean) |
| Wei, J.L., et al., *Associations of soybean products intake with blood pressure changes and hypertension incidence: the China-PAR project.* Journal of Geriatric Cardiology, 2020. **17**(7): p. 384-392. | wrong exposure/intervention (soy products) |
| Wilunda, C., et al., *Soy food and isoflavones are not associated with changes in serum lipids and glycohemoglobin concentrations among Japanese adults: a cohort study.* European Journal of Nutrition, 2020. **59**(5): p. 2075-2087. | wrong exposure/intervention |
| Wolfe, B.M. and P.M. Giovannetti, *Elevation of VLDL-cholesterol during substitution of soy protein for animal protein in diets of hypercholesterolemic Canadians.* Nutrition Reports International, 1985. **32**(5): p. 1057-1065. | wrong exposure/intervention |
| Xue et al. Association of soy food with cardiovascular outcomes and all-cause mortality in a Chinese population: a nationwide prospective cohort study | wrong exposure/intervention (no separate analysis for soybean) |
| Yamaoka, S., et al., *Risk reduction of lifestyle-related diseases in young adults on soy- or fish-rich traditional Japanese meals.* Clinical and experimental pharmacology & physiology, 2007. **34**: p. S79‐S81. | wrong exposure/intervention (legume protein/powder) |
| Yamashita, Y., et al., *Black Soybean Improves Vascular Function and Blood Pressure: A Randomized, Placebo Controlled, Crossover Trial in Humans.* Nutrients, 2020. **12**(9): p. 10. | wrong exposure/intervention (not only beans differed) |
| Yang, G., et al., *Longitudinal study of soy food intake and blood pressure among middle-aged and elderly Chinese women.* American Journal of Clinical Nutrition, 2005. **81**(5): p. 1012-1017. | wrong exposure/intervention (soy protein) |
| Yildirir, A., et al., *Soy protein diet significantly improves endothelial function and lipid parameters.* Clinical Cardiology, 2001. **24**(11): p. 711-716. | wrong exposure/intervention |
| Zhang, X., et al., *Soy food consumption is associated with lower risk of coronary heart disease in Chinese women.* Journal of Nutrition, 2003. **133**(9): p. 2874-2878. | wrong exposure/intervention |
| Zhu, N., et al., *Adherence to a healthy lifestyle and all-cause and cause-specific mortality in Chinese adults: a 10-year prospective study of 0.5 million people.* International Journal of Behavioral Nutrition & Physical Activity, 2019. **16**(1): p. 98. | wrong exposure/intervention |
| Bahls, L.D., et al., *Evaluation of the intake of a low daily amount of soybeans in oxidative stress, lipid and inflammatory profile, and insulin resistance in patients with metabolic syndrome.* Arquivos Brasileiros de Endocrinologia e Metabologia, 2011. **55**(6): p. 399-405. | wrong language |
| Becerra-Tomas, N., et al., *Replacing red meat and processed red meat for white meat, fish, legumes or eggs is associated with lower risk of incidence of metabolic syndrome.* Clinical Nutrition, 2016. **35**(6): p. 1442-1449. | wrong outcome (MetS) |
| Borgi, L., et al., *Fruit and Vegetable Consumption and the Incidence of Hypertension in Three Prospective Cohort Studies.* Hypertension, 2016. **67**(2): p. 288-293. | wrong outcome (observational analysis, hypertension) |
| Chen, G.C., et al. *Adherence to Recommended Eating Patterns Is Associated With Lower Risk of Peripheral Arterial Disease: Results From the Women's Health Initiative*. Hypertension, 2021. **78**(2): p. 447-455. | wrong outcome (PAD) |
| Feskens, E.J., C.H. Bowles, and D. Kromhout, *Carbohydrate intake and body mass index in relation to the risk of glucose intolerance in an elderly population.* American Journal of Clinical Nutrition, 1991. **54**(1): p. 136-140. | wrong outcome |
| Golzarand, M., et al., *Protein Foods Group and 3-Year Incidence of Hypertension: A Prospective Study From Tehran Lipid and Glucose Study.* Journal of Renal Nutrition, 2016. **26**(4): p. 219-225. | wrong outcome (observational analysis, hypertension) |
| Guo, F., et al., *Legume consumption and risk of hypertension in a prospective cohort of Chinese men and women.* British Journal of Nutrition, 2020. **123**(5): p. 564-573. | wrong outcome (observational analysis, hypertension) |
| Mirmiran, P., et al., *Dietary Intake, Changes in Lipid Parameters and the Risk of Hypertriglyceridemia: A Prospective Approach in the Tehran Lipid and Glucose Study.* International Journal for Vitamin & Nutrition Research, 2014. **84**(5): p. 269-276. | wrong outcome |
| Riseberg et al. Specific Dietary Protein Sources Are Associated with Cardiometabolic Risk Factors in the Boston Puerto Rican Health Study | wrong outcome (observational analysis, CVD risk factors) |
| Shirai et al. Association between functional foods and cardiometabolic health in a real-life setting: a longitudinal observational study using objective diet records from an electronic purchase system | wrong outcome (observational analysis, CVD risk factors) |
| Zhu et al. Adherence to a Plant-Based Diet and Consumption of Specific Plant Foods-Associations with 3-Year Weight-Loss Maintenance and Cardiometabolic Risk Factors: A Secondary Analysis of the PREVIEW Intervention Study | wrong outcome (observational analysis, CVD risk factors) |
| Bakhtiaria, A., et al., *The HOMA-IR and lipid profile in response to isoflavones in elderly women with the metabolic syndrome.* Maturitas, 2012. **71**: p. S35. | wrong publication type |
| Bell, R.C., et al., *Dried beans lower cholesterol and glycated hemoglobin while peas lower blood pressure in adults with mild hypercholesterolemia.* FASEB journal, 2017. **31**(1). | wrong publication type |
| de Koning, L. and S.S. Anand, *Vascular viewpoint.* Vascular Medicine, 2004. **9**(2): p. 145-146. | wrong publication type |
| Friihbeck, G., I. Monreal, and S. Santidriân, *Hyfocholesterolemic effect of field bean vicia faba u intake in hyperchotesterolemic undergraduate students.* FASEB Journal, 1996. **10**(3). | wrong publication type |
| Gravel, K., et al., *Does legumes consumption in a real life context can improve components of metabolic syndrome? A randomized controlled trial.* Journal of diabetes, 2009. **1**: p. A277‐. | wrong publication type |
| Hanifi, A., et al., *Health benefits of a pulse-based diet for soccer players during regular season play.* FASEB journal, 2015. **29**(1). | wrong publication type |
| Hill, A.M., et al., *A 12-week randomised controlled trial to evaluate effects of dietary pulse consumption on cardiovascular disease risk factors.* FASEB journal, 2017. **31**(1). | wrong publication type |
| Joshi, S.S., A. Mehta, and S. Joshi, *Effect of kidney beans on blood glucose levels in adults with prediabetes.* Diabetes., 2014. **63**: p. A193. | wrong publication type |
| Steinberg, F. and A. Villablanca, *Soy proteins decrease lipid and lipoprotein levels in pre- and post-menopausal women.* FASEB Journal, 1998. **12**(5). | wrong publication type |
| Zahradka, P., et al., *Improvements in arterial stiffness due to bean and pea consumption are determined by metabolic state.* FASEB journal, 2017. **31**(1). | wrong publication type |
| Al-Shaar, L., et al., *Red meat intake and risk of coronary heart disease among US men: prospective cohort study.* Bmj, 2020. **371**: p. m4141. | wrong study design |
| Anil, S., et al., *Identification of dietary patterns associated with blood pressure in a sample of overweight Australian adults.* Journal of Human Hypertension, 2016. **30**(11): p. 672-678. | wrong study design |
| Duane, W.C., *Effects of soybean protein and very low dietary cholesterol on serum lipids, biliary lipids, and fecal sterols in humans.* Metabolism: Clinical & Experimental, 1999. **48**(4): p. 489-494. | wrong study design |
| Garcia-Palmieri, M.R., et al., *Relationship of dietary intake to subsequent coronary heart disease incidence: The Puerto Rico Heart Health Program.* American Journal of Clinical Nutrition, 1980. **33**(8): p. 1818-1827. | wrong study design |
| Ibsen, D.B., et al., *Replacement of red and processed meat with other food sources of protein and the risk of type 2 diabetes in European populations: The epic-interact study.* Diabetes Care, 2020. **43**(11): p. 2660-2667. | wrong study design |
| Matthan, N.R., et al., *Effect of soy protein from differently processed products on cardiovascular disease risk factors and vascular endothelial function in hypercholesterolemic subjects.* American Journal of Clinical Nutrition, 2007. **85**(4): p. 960-966. | wrong study design |
| Menotti, A., et al., *Food intake patterns and 25-year mortality from coronary heart disease: cross-cultural correlations in the Seven Countries Study. The Seven Countries Study Research Group.* European Journal of Epidemiology, 1999. **15**(6): p. 507-515. | wrong study design |
| Oldewage-Theron, W. and A. Egal, *The effect of consumption of soy foods on the blood lipid profile of women: a pilot study from Qwa-Qwa.* Journal of Nutritional Science & Vitaminology, 2013. **59**(5): p. 431-436. | wrong study design |
| Rosa, M.L., et al., *Brazil's staple food and incident diabetes.* Nutrition, 2014. **30**(3): p. 365-368. | wrong study design (retrospective cohort study) |
| Ruiz Esparza Cisneros, J., et al., *Effect of dietary intervention with a legume-based food product on malondialdehyde levels, HOMA index, and lipid profile.* Endocrinologia Diabetes y Nutricion, 2020. **67**(4): p. 235-244. | wrong study design |
| van Nielen, M., et al., *Partly replacing meat protein with soy protein alters insulin resistance and blood lipids in postmenopausal women with abdominal obesity.* Journal of Nutrition, 2014. **144**(9): p. 1423-1429. | wrong study design |
| Welty, F.K., et al., *Effect of soy nuts on blood pressure and lipid levels in hypertensive, prehypertensive, and normotensive postmenopausal women.* Archives of Internal Medicine, 2007. **167**(10): p. 1060-1067. | wrong study design |
| Wurtz, A.M.L., et al., *Replacing the consumption of red meat with other major dietary protein sources and risk of type 2 diabetes mellitus: a prospective cohort study.* American Journal of Clinical Nutrition, 2021. **113**(3): p. 612-621. | wrong study design |
| Zahradka, P., et al., *Daily non-soy legume consumption reverses vascular impairment due to peripheral artery disease.* Atherosclerosis, 2013. **230**(2): p. 310-314. | wrong study design |
| Zarrazquin, I., et al., *Longitudinal study: lifestyle and cardiovascular health in health science students.* Nutricion Hospitalaria, 2014. **30**(5): p. 1144-1151. | wrong study design |

**Supplementary table 3.** Summary of findings from observational studies.

| **Article, reference** | **Exposure details** | **No. of exposure assessments** | **Exposure assessment** | **Exposure amounts** | **Person-years** | **Outcome (No. of events)** | **Outcome assessment** | **Confounders** | **Significant association** | |
| --- | --- | --- | --- | --- | --- | --- | --- | --- | --- | --- |
| ***Cardiovascular outcomes*** | | | | | | | | | | |
| Bernstein 2012 ([78](#_ENREF_78)) | Legumes (beans, peas, soybeans, tofu) | 6 | FFQ | Median per quintile (servings/d): 0.07, 0.14, 0.18, 0.27, 0.48 | 2875339 | Stroke incidence (4030) | Medical records or death certificates | age, timeperiod, BMI, smoking, physical activity, family history of early myocardial infarction, menopausal status, multivitamins, aspirins, energy intake, intake of fiber, alcohol, trans fatty acids, fruit, vegetables, protein sources | None |  |
| Blekkenhorst 2017 ([79](#_ENREF_79)) | Legumes (beans, peas, soybeans, tofu) | 3 | FFQ | Mean±SD (g/d): 27.0±18.9  *Not enough data to include in high vs. low or dose-response analysis.* | 15947 | CVD mortality (238) | Death certificate | age, BMI, physical activity, alcohol, smoking, socioeconomic status, calcium supplementation group, medication, glomerular filtration rate, energy intake | None |  |
| Farvid 2017 ([80](#_ENREF_80)) | Legumes (beans, lentils, peas, soybeans) | 1 | FFQ | Median per quintile (servings/d): 0.03, 0.08, 0.12, 0.17, 0.26 | 339867 | CVD mortality (1467), CHD mortality (764), Stroke mortality (507) | Family-reported, medical documents checked | age, sex, ethnicity, education, marital status, residency, smoking, opium use, alcohol, BMI, systolic blood pressure, physical activity, family history of cancer, wealth score, medication, energy intake | None |  |
| Fraser 1992 ([81](#_ENREF_81)) | Legumes (beans, lentils, peas) | 1 | FFQ | Times/week (% of participants): <1 (40%), 1-2 (37%), >2 (23%) | Not reported | CHD incidence (394) | Medical records or death certificates | age, sex, smoking, physical activity, weight, high blood pressure | None |  |
| Fung 2018 ([82](#_ENREF_82)) | Legumes (beans, peas, soybeans, tofu) | 8 (NHS), 5 (NHS II), 6 (HPFS) | FFQ | Mean across diet score tertiles ranging from 0.1 to 0.4 servings/d  *Not enough data to include in dose-response analysis.* | Not reported | CHD incidence (7161) | Medical records or death certificates | age, cohort, energy intake, smoking, alcohol, physical activity, BMI, family history of myocardial infarction, multivitamins, aspirin, baseline history of diabetes, hypertension or hyperlipidemia, menopausal status, postmenopausal hormone use (NHS), oral contraceptive use (NHS II), selected food groups | None |  |
| Golzarand 2022 ([43](#_ENREF_43)) | Legumes (beans, lentils, soybeans) | 1 | FFQ | Assessed as meals/week: <1, 1-3, >3  *Not enough data to include in high vs. low or dose-response analysis.* | 6986 | CVD incidence (200) | Medical records or death certificates | age, sex, BMI, smoking, socioeconomic status, energy intake, diabetes, hypertension | ↓ | Highest vs. lowest legume intake associated with lower CVD risk in fully adjusted model, HR (95% CI): 0.35 (0.19, 0.62) |
| Haring 2014 ([83](#_ENREF_83)) | Legumes | 2 | FFQ | Median per quintile (serving/d): 0.07, 0.14, 0.21, 0.28, 0.57 | 233687 | CHD incidence (1147) | Medical records or death certificates | age, sex, race, study center, energy intake, smoking, education, systolic blood pressure, antihypertensive medications, HDL cholesterol, total cholesterol, lipid lowering medications, BMI, waist-to-hip ratio, alcohol, physical activity, intake of carbohydrate, fiber, fat, magnesium | None |  |
| Haring 2015 ([84](#_ENREF_84)) | Legumes | 2 | FFQ | Median per quintile (serving/d): 0.07, 0.14, 0.21, 0.28, 0.57 | 236045 | Stroke incidence (699) | Medical records or death certificates | age, sex, race, study center, energy intake, smoking, education, systolic blood pressure, antihypertensive medications, HDL cholesterol, total cholesterol, lipid lowering medications, BMI, waist-to-hip ratio, alcohol, physical activity, intake of carbohydrate, fiber, fat, magnesium | None |  |
| Im 2021 ([31](#_ENREF_31)) | Soybeans | 2 | FFQ | Median per quartile (servings/week), premenopausal/postmenopausal: 1.0/1.2, 2.2/2.4, 3.6/4.0, 7.7/9.8 | 34962 | CVD incidence (282) | Self-report | age, residental area, household income, smoking, alcohol, physical activity, BMI, intake of meat, fish/seafood, fruits, vegetables, dietary supplements, fat, cholesterol, history of hypertension, dyslipidemia and diabetes | None |  |
| Kokubo 2007 ([27](#_ENREF_27)) | Soybeans | 2 | FFQ | Exposure categories (days/week): 0, 1-2, ≥3 | 503998 | CHD incidence (308), Stroke incidence (587), CVD mortality (232) | Medical records or death certificates | age, sex, smoking, alcohol, BMI, history of hypertension or diabetes, hypercholesterolemia medications, education, physical activity, intake of fruits, vegetables, fish, salt, energy intake, menopausal status (females), pulic health centre | None |  |
| Martinez-Gonzales 2011 ([85](#_ENREF_85)) | Legumes | 1 | FFQ | Assessed for <21 g/d vs. ≥21 g/d  *Not enough data to include in high vs. low or dose-response analysis.* | 66577 | CVD incidence (100), CHD incidence (68) | Self-report, confirmed by medical records or death certificates | age, sex, family history of CHD, energy intake, physical activity, smoking, BMI, baseline diabetes, aspirin, history of hypertension or hypercholesterolemia | None |  |
| Miller 2017 ([37](#_ENREF_37)) | Legumes (beans, lentils, peas) | 1 | FFQ | Assessed as servings/time unit: <1/month, 1/month to <1/week, 1/week to <3/week, 3/week to <1/day, ≥1/day | Not reported | CVD incidence (4784), CHD incidence (2143), Stroke incidence (2234), CVD mortality (1649) | Not reported | age, sex, energy intake, smoking, urban or rural, physical activity, baseline diabetes, education, intake of white meat, red meat, bread, cereal, study centre | None |  |
| Mizrahi 2009 ([33](#_ENREF_33)) | Legumes | 3 | Diet history | Range per quartile (g/d), female/male: 0-1/0-2, 2-3/3-5, 4-6/6-9, 7-43/10-101 | approx. 78000 | Stroke incidence (625) | Medical records or death certificates | age, sex, BMI, smoking, physical activity, serum cholesterol, blood pressure, energy intake | ↓ | Highest vs. lowest legume quartile associated with lower risk of ischemic stroke in fully adjusted model, RR (95%) CI: 0.72 (0.54, 0.96) |
| Nagura 2009 ([28](#_ENREF_28)) | Soybeans | 1 | FFQ | Mean per tertile (servings/week): 0.8, 1.8, 3.0 | 756054 | CVD mortality (2243), CHD mortality (452), Stroke mortality (1053) | Death certificate | age, sex, BMI, smoking, alcohol, physical activity, education, perceived mental stress, history of hypertension or diabetes, dietary cholesterol, saturated fatty acids, n-3 PUFA, sodium | ↓ | Highest vs. lowest soybean intake associated with lower CVD mortality in fully adjusted model, HR (95% CI): 0.84 (0.74-0.95) |
| Nouri 2021 ([44](#_ENREF_44)) | Legumes (beans, lentils, peas, soybeans) also subgroups: soybeans, non-soybeans (beans, lentils, peas) | 3 | FFQ | Assessed as meals/week: <1, 1-3, >3 | Not reported | CVD incidence (751) | Medical records or death certificates | age, sex, education, marital status, smoking, dietary score, physical activity, BMI, anti-dyslipidemia, antihypertensive, antidiabetic medications | ↓ | Highest vs. lowest total legume and soybean intake associated with lower CVD risk in fully adjusted models, HR (95% CI): 0.805 (0.650-0.998), and 0.815 (0.673-0.988) for total legumes and soybeans, respectively |
| Papandreou 2019 ([45](#_ENREF_45)) | Legumes (beans, lentils, peas) also subgroups: lentils, chickpeas, dry beans, fresh peas | 6 | FFQ | Median per tertile (g/d): 14, 20, 27 | 42464 | CVD mortality (103) | Medical records or death certificates | age, sex, intervention group, diabetes, hypertension, hypercholesterolemia, BMI, smoking, education, physical activity, antihypertensive medication, antidiabetic agents, statins, alcohol, diet score | ↑ | Highest vs. lowest legume and dry beans intake associated with higher CVD mortality, HR (95% CI) 1.72 (1.02, 2.89) and 2.23 (1.32, 3.78) for total legume and dry beans, respectively |
| Perez-Cornago 2021 ([36](#_ENREF_36)) | Legumes (beans, lentils, peas) | 1 | FFQ or diet history | Median per quintile (g/d): 0.0, 2.4, 8.1, 16.6, 45.7 | 6170299 | CHD incidence (8504) | Self-report and medical records or death certificates | age, sex, study centre, smoking, history of diabetes, hypertension or hyperlipidemia, physical activity, employment, education, alcohol, BMI, energy intake, red and processed meat, cheese | None |  |
| Stefler 2017 ([86](#_ENREF_86)) | Legumes | 1 | FFQ | Median per tertile (g/d): 3.5, 14.0, 31.9 | Not reported | CVD mortality (438), CHD mortality (226), Stroke mortality (109) | Death certificate | age, sex, cohort, education, marital status, household amenities score, smoking, physical activity, energy intake, vitamin supplements | None |  |
| Tong 2020 ([35](#_ENREF_35)) | Legumes (beans, lentils, peas) | 1 | mainly FFQ | Median per quintile (g/d): 0.0, 2.4, 8.1, 16.6, 45.7 | 4145676 | Stroke incidence (7378) | Self-report and medical records or death certificates | age, sex, study centre, smoking, history of diabetes, hypertension or hyperlipidemia, physical activity, employment, education, alcohol, BMI, energy intake, cereal fibre, fruit, vegetables, milk, yogurt, cheese, red meat | None |  |
| Yamasaki 2015 ([29](#_ENREF_29)) | Soybeans | 1 | FFQ | Exposure categories (% of participants in each category): rarely (8%), 1-2 times/month (24%), 1-2 times/week (33%), 3-4 times/week (21%), almost daily (13%) | 50822 | CVD mortality (213) | Death certificate | age, sex, BMI, HDL cholesterol, BMI, alcohol, smoking, hypertension, diabetes, education, menopause (females) | None |  |
| Yu 2014 ([87](#_ENREF_87)) | Legumes (beans, peas, soybeans) | 1 | FFQ | Median per quartile (g/d), female/male: 8.5/10.8, 17.7/22.8, 28.5/35.8, 50.7/62.8 | Not reported | CHD incidence (365) | Medical records or death certificates | age, sex, birth cohort, energy intake, BMI, income, education, smoking, alcohol, physical activity, aspirin, vitamin E, multivitamin supplements, menopause and hormone replacement therapy (females), intakes of red meat and fish/shellfish, history of diabetes, hypertension, dyslipidemia | None |  |
| ***Type 2 diabetes*** | | |  |  |  |  |  |  |  |  |
| Bazzano 2008 ([49](#_ENREF_49)) | Legumes (beans, peas, soybeans, tofu) | 6 | FFQ | Median in lowest, middle and highest quintiles (servings/d): 0.07, 0.17, 0.45 | 1203994 | T2D (4529) | Self-report, confirmed by symptom evaluation, reported elevated plasma glucose or use of oral hypoglycemic medication or insulin | age, BMI, physical actvity, family history of diabetes, postmenopausal hormones, alcohol, smoking, energy intake, whole grains, nuts, processed meats, coffee, potatoes, sugar-sweetened soft drinks | ↑ | Highest vs. lowest intake associated with higher diabetes risk, HR (95% CI): 1.14 (1.03, 1.25) |
| Becerra-Tomás 2018 ([47](#_ENREF_47)) | Legumes (beans, lentils, peas) also subgroups: lentils, chickpeas, dry beans, fresh peas | 4 | FFQ | Median per quartile (g/d): 12.7, 17.6, 22.0, 28.8 | 13797 | T2D (266) | Medical records or fasting glucose values | age, sex, intervention group, alcohol, smoking, education, physical activity, baseline hypertension, hypercholesterolemia, antihypertensive medications, lipid-lowering drugs, baseline fasting plasma glucose, diet score, BMI | ↓ | Highest vs. lowest total legume intake and lentil intake associated with lower diabetes risk, HR (95% CI): 0.65 (0.43-0.96) and 0.67 (0.46-0.98) for total legumes and lentils, respectively |
| Ericson 2013 ([34](#_ENREF_34)) | Legumes | 1 | Diet history | Median per quartile (g/d), female/male: 0/0, 13/18, 20/24, 26/38, 49/61 | 320703 | T2D (1709) | Medical records | age, sex, BMI, time-period, season, energy intake, education, smoking, alcohol, physical activity, BMI | None |  |
| Hodge 2004 ([38](#_ENREF_38)) | Legumes (beans, peas, lentils) | 1 | FFQ | Cut points per quartile (times/week): <2.0, 2.0–3.4, 3.5–4.9, ≥5.0 | Not reported | T2D (365) | Self-report, confirmed by medical doctors | age, sex, birth country, physical activity, family history of diabetes, alcohol, education, 5-year weight change, energy intake | None |  |
| Khalili-Moghadam 2018 ([46](#_ENREF_46)) | Legumes | 1 | FFQ | Mean per diet score tertiles (servings/d): 0.08, 0.15, 0.20 | Not reported | T2D (143) | Antidiabetic drug use, 2-h plasma glucose ≥200 mg/dL or fasting plasma glucose ≥126 mg/dL | diabetes risk score | ↓ | Highest vs. lowest legume intake associated with lower diabetes risk in fully adjusted models, HR (95% CI): 0.56 (0.35-0.89) |
| Liu 2004 ([88](#_ENREF_88)) | Legumes | 1 | FFQ | Median per quintile (servings/d): 0.13, 0.21, 0.29, 0.50, 0.86 | 332906 | T2D (1614) | Self-report | age, energy intake, BMI, smoking, physical activity, history of hypertension or high cholesterol, family history of diabetes | None |  |
| Meyer 2000 ([89](#_ENREF_89)) | Legumes (mature beans) | 1 | FFQ | Median per quintile (servings/week): 1.0, 2.0, 2.5, 4.0, 6.5 | 202653 | T2D (1141) | Self-report | age, energy intake, physical activity, BMI, waist-to-hip ratio, smoking, alcohol, education, family history of diabetes | None |  |
| O'Connor 2020 ([48](#_ENREF_48)) | Legumes (beans, peas, lentils) | 2 | FFQ | *Considered the same as in Haring 2014 and 2015 (all from the ARIC cohort):*  *Median per quintile (serving/d): 0.07, 0.14, 0.21, 0.28, 0.57*  *Not enough data to include in high vs. low or dose-response analysis.* | Not reported | T2D (4024) | Self-report or fasting plasma glucose ≥126 mg/dL or non-fasting plasma glucose ≥200 mg/dL | age, sex, energy intake, race-centre, education, smoking, physical activity, fasting glucose, hypertension, LDL cholesterol, BMI, family history of diabetes | ↓ | Higher component scores for legumes, representing intake above sex-specific medians, were inversely associated with diabetes risk, HR (95% CI): 0.92 (0.86-0.98) |
| Villegas 2008 ([32](#_ENREF_32)) | Legumes (beas, peas, lentils), soybeans | 2 | FFQ | Median per quintile (g/d):  Legumes (beans, peas, lentils): 5.6, 10.7, 15.5, 22.3, 37.1,  Soybeans: 2.8, 6.6, 11.1, 17.3, 32.0  *Legumes and soybeans combined in meta-analysis* | 297744 | T2D (1608) | Self-reported, confirmed by blood measurements or use of hypoglycemic drugs | age, energy intake, BMI, waist-to-hip ratio, smoking, alcohol, vegetable intake, fiber, physical activity, income, education, occupation, hypertension | ↓ | Highest vs. lowest legume (beans, peas, lentils) and soybeans intake associated with lower diabetes risk, RR (95% CI): 0.76 (0.64, 0.90) and 0.53 (0.45, 0.62) for legumes and soybeans, respectively |
| Yan 2021 ([30](#_ENREF_30)) | Soybeans | 1 | FFQ | Exposure categories (% of participants in each category): less than weekly (60%), 1-2 times/week (24%), ≥3 times/week (16%) | Not reported | T2D (593) | Self-reported, compared with glucose concentrations or treatment history in a subgroup | age, sex, area, energy intake, BMI, history of hypertension, family history of diabetes, physical activity, alcohol, education, sleep, smoking, mental stress, work status, coffee, green tea, rice, tofu, miso soup | None |  |

Footnotes:

Abbreviations: BMI, body mass index; CHD, coronary heart disease; CI, confidence interval; CVD, cardiovascular disease; d, day; HR, hazard ratio; RR, risk ratio; SD, standard deviation; T2D, type 2 diabetes

**Supplementary table 4.** Summary of findings from intervention studies.

| **Article, reference** | **Exposure assessment** | **Intervention details and amounts** | **Control** | **Co-intervention** | **Outcomes assessed** | **Significant changes** | **Significant results** |
| --- | --- | --- | --- | --- | --- | --- | --- |
| Abeysekara 2012 ([50](#_ENREF_50)) | FFQ and legume log | Mixed legumes 150/250 g/d dry/cooked weight (green lentils, red split lentils, chickpeas, yellow split peas, and pinto, fava, broad, black and kidney beans) | Usual diet |  | TC, HDL-C, LDL-C, TG, glucose, insulin | ↓TC ↓ LDL-C | Compared with control, the intervention decreased TC by 8.3% and LDL-C by 7.9% (also seen in subanalysis of individuals with high baseline lipid levels). Difference (95% CI) between change in intervention and change in control diet: TC -0.38 (-0.59,-0.17) mmol/L, LDL-C -0.23 (-0.43, -0.03) mmol/L. |
| Azadbakht 2007 ([51](#_ENREF_51)) | 3-d food record | Soy-nut 30 g/d (DASH diet, but red meat replaced with 30 g soy-nut/serving of red meat) | Control diet (DASH) | DASH diet | TC, HDL-C, LDL-C, TG, glucose, insulin, HOMA-IR, SBP, DBP | ↓TC ↓ LDL-C  ↓ HOMA-IR | The intervention diet reduced HOMA-IR (-12.9±0.9, p<0.01), fasting plasma glucose (-5.1±0.6%, p<0.01) and LCL-C (-9.5±0.6%, p<0.01) more than did control. |
| Bakhtiari 2019 ([52](#_ENREF_52)) | 3-d food record | Soy-nut 35 g/d | Usual diet |  | TC, HDL-C, LDL-C, TG, glucose, insulin, HOMA-IR | ↓TC,  ↓LDL-C, ↓glucose, ↓insulin, ↓HOMA-IR | Mean changes in soy-nut group from baseline to end of trial, that were significantly different compared with control: TC -29.2±3.6 mg/dl, LDL-C -23.1±2.0 mg/dl, glucose -14.4±1.1 mg/dl, insulin -2.0±0.3 µIU/ml, HOMA-IR -0.9±0.1 |
| Cobiac 1990 ([90](#_ENREF_90)) | 3-d weighed food record | Baked beans 377 g/d (six 440 g cans per week) | Usual diet + spaghetti 377 g/d |  | TC, HDL-C, LDL-C, TG, glucose | None |  |
| Doma 2021 ([53](#_ENREF_53)) | 3-d food record and legume log | Beans (canned blacked, navy, pinto, dark red kidney, white kidney): 180 g/d (1CB) or 90 g/d (½CB) cooked weight  *1CB used in meta-analysis* | Cooked white rice 132 g/d |  | TC, HDL-C, LDL-C, TG, glucose, insulin, HOMA-IR | ↓TC (1CB l),  ↓LDL-C (1CB) | TC at end of trial was lower for 1CB (P = 0.04) but not ½CB (P = 0.77) compared with control (-5.46%, -2.74%, -0.65% changes from study day 1, respectively) and did not differ between 1CB and ½CB (P = 0.17). LDL-C at end of trial was also lower for 1CB (P = 0.002) but not ½CB (P = 0.30) compared with control (-8.08%, -3.84%, +0.49% changes from study day 1, respectively) and did not differ between 1CB and ½CB (P = 0.11). |
| Duane 1997 ([57](#_ENREF_57)) | Meals provided by hospital | Mixed legumes 120 g/d dry weight | Control diet (hospital diet) | Hospital diet | TC, HDL-C, LDL-C, TG | ↓ LDL-C | Mean serum LDL-C was significantly lower during legume consumption compared to control (126 vs. 138 mg/dl, P = 0.039). |
| Lin 1981 ([54](#_ENREF_54)) | Unclear | Beans 25-100 g/d (50-100 g whole beans everyday or every second day) | No beans |  | TC, TG  *Not included in meta-analysis* | ↓ TC | TC in intervention group at start vs. end of trial: 258.3±50.3 mg% vs. 221.9±39.8 mg% (p<0.001). TC in control group at start vs. end of trial: 234.5±39.9 mg% vs. 232.2±46.4 mg%, non-significant. |
| Mackay 1992 ([39](#_ENREF_39)) | FFQ, 4-d food record, 3-d food record | Beans 80 g/d cooked weight | Other intervention diets: Low-fibre oat bran; High-fibre oat bran  *Low-fibre oat bran used as control in meta-analysis* | Moderately low-fat diet | TC, HDL-C, LDL-C, TG | Compared to run-in: ↑ HDL-C | HDL-C was significantly higher after intervention diets than run-in (1.27±0.27 mmol/L vs. 1.15±0.22 mmol/L, p<0.05) |
| Mizelman 2020 ([59](#_ENREF_59)) | 24-h recalls | Mixed pulses 156/260 g/d dry/cooked weight (lentils, chickpeas, beans, split peas) | Usual diet | Lentil based bars or high glycemic index bars | TC, HDL-C, insulin | ↑ HDL-C in women | Women increased HDL on the intervention compared to control diet (0.5±0.7 mmol/L vs. -0.6±0.3 mmol/L, p < 0.01) . |
| Nestel 2004 ([40](#_ENREF_40)) | FFQ | Chickpeas 140 g/d cooked weight + bread and biscuits baked with chickpea flour | Whole-grain wheat-based diet (bread, breakfast cereals, shortbread biscuits) |  | glucose, insulin, HOMA-IR | None |  |
| Pittaway 2006 ([41](#_ENREF_41)) | 4-d weighed FR | Chickpeas 140 g/d cooked weight + bread and biscuits baked with chickpea flour | Whole-grain wheat-based diet (bread, breakfast cereals, shortbread biscuits) | Requested to refrain from other legumes, cholesterol lowering foods or foods with high-fibre claims | TC, HDL-C, LDL-C, TG | ↓TC ↓ LDL-C | TC and LDL-C were significantly lower (both p<0.01) by 3.9% and 4.6%, respectively, after intervention compared with control. |
| Pittaway 2007 ([42](#_ENREF_42)) | 4-d weighed FR | Chickpeas 140 g/d cooked weight + bread and biscuits baked with chickpea flour | Whole-grain wheat-based diet (bread, breakfast cereals, shortbread biscuits) | Some participants commenced a third lower-fibre wheat-based dietary intervention (lower-fibre diet) of three weeks duration | glucose, insulin, HOMA-IR, (TC, HDL-C, TG results reported in Pittaway 2006) | None for glucose, insulin, HOMA-IR |  |
| Saraf-Bank 2016 ([91](#_ENREF_91)) | 24-h recall | Mixed legumes 37 g/d dry weight (pinto beans and lentils) | Usual diet | Asked to adhere to general diet and PA recommendations | TC, LDL-C, HDL-C, TG, glucose, SBP, DBP | No effects |  |
| Tischmann 2022 ([58](#_ENREF_58)) | FFQ | Soy-nut 67 g/d | Usual diet (no soy) | No dietary supplements | TC, HDL-C, LDL-C, TG | ↓LDL-C | Serum LDL-cholesterol was lower after the intervention as compared with the control period, treatment effect, mean difference (95% CI): -0.17 mmol/L (-0.32, -0.02), p = 0.027. |
| Winham 2007a ([56](#_ENREF_56)) | 2-d/24h diet records | Pinto group: 130 g/d of pinto beans  Peas group: 130 g/d of black-eyed peas  *Intervention groups pooled in meta-analysis* | Usual diet + canned carrots 130 g/d |  | TC, LDL-C, HDL-C, TG, glucose, insulin, HOMA-IR | Pinto compared to control: ↓ TC ↓ LDL-C | A significant treatment-by-time effect impacted serum TC (p=0.026) and LDL-C (p=0.033) after the intervention, Paired t-tests indicated that pinto beans were responsible for this effect (p=0.003; p=0.008). Mean change from pre-intervention to post-intervention of serum TC was for -19±5 mg/dL for pinto bean, 2.5±6 mg/dl for black-eyed pea and 1±5 mg/dl for control (p = 0.011). Mean change from pre-intervention to post-intervention of serum LDL-C was -14±4 mg/dl for pinto bean, 4±5 mg/dl for black-eyed pea and 1±4 mg/dl for control (p = 0.013). Pinto beans differed significantly from control (p = 0.021). |
| Winham 2007b ([55](#_ENREF_55)) | 2-d/24h diet records | Baked beans 130 g/d cooked weight (canned navy beans) | Usual diet + canned carrots 130 g/d |  | TC, LDL-C, HDL-C, TG, glucose, insulin, HOMA-IR | ↓ TC | Mean percentage change of serum TC for baked beans was −5.6% ± 1.5% SEM in contrast to 0.5% ± 1.8% SEM for the control (p=0.01). |

Footnotes:

Abbreviations: HDL-C, high density lipoprotein cholesterol; HOMA-IR, homeostatic model assessment for insulin resistance; LDL-C, low density lipoprotein cholesterol; TC, total cholesterol; TG, triglycerides


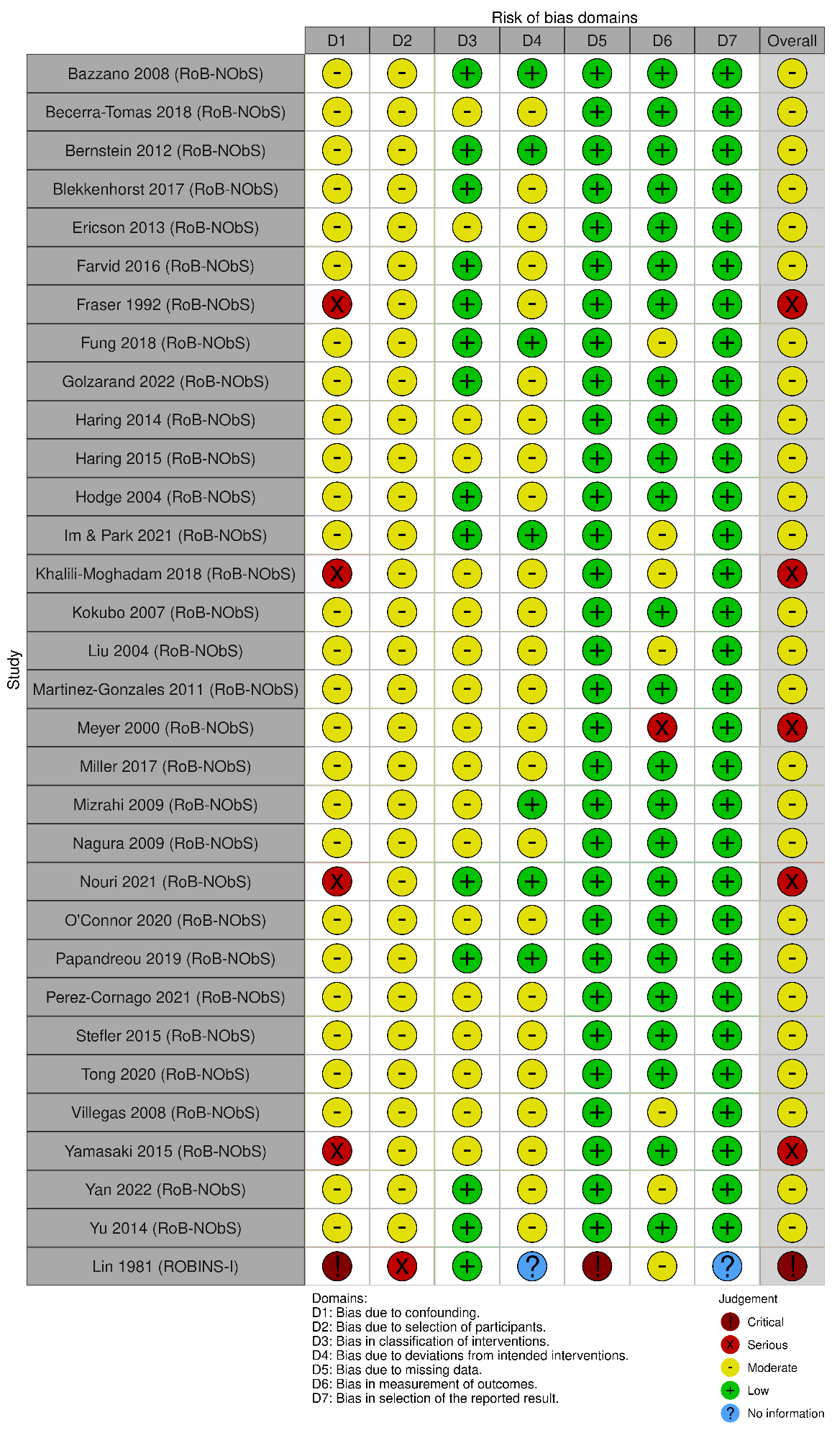


**Supplementary figure 1.** Risk of bias per domain in observational studies (D3 and D4 refer to exposure, not interventions) and the single non-randomized intervention trial.


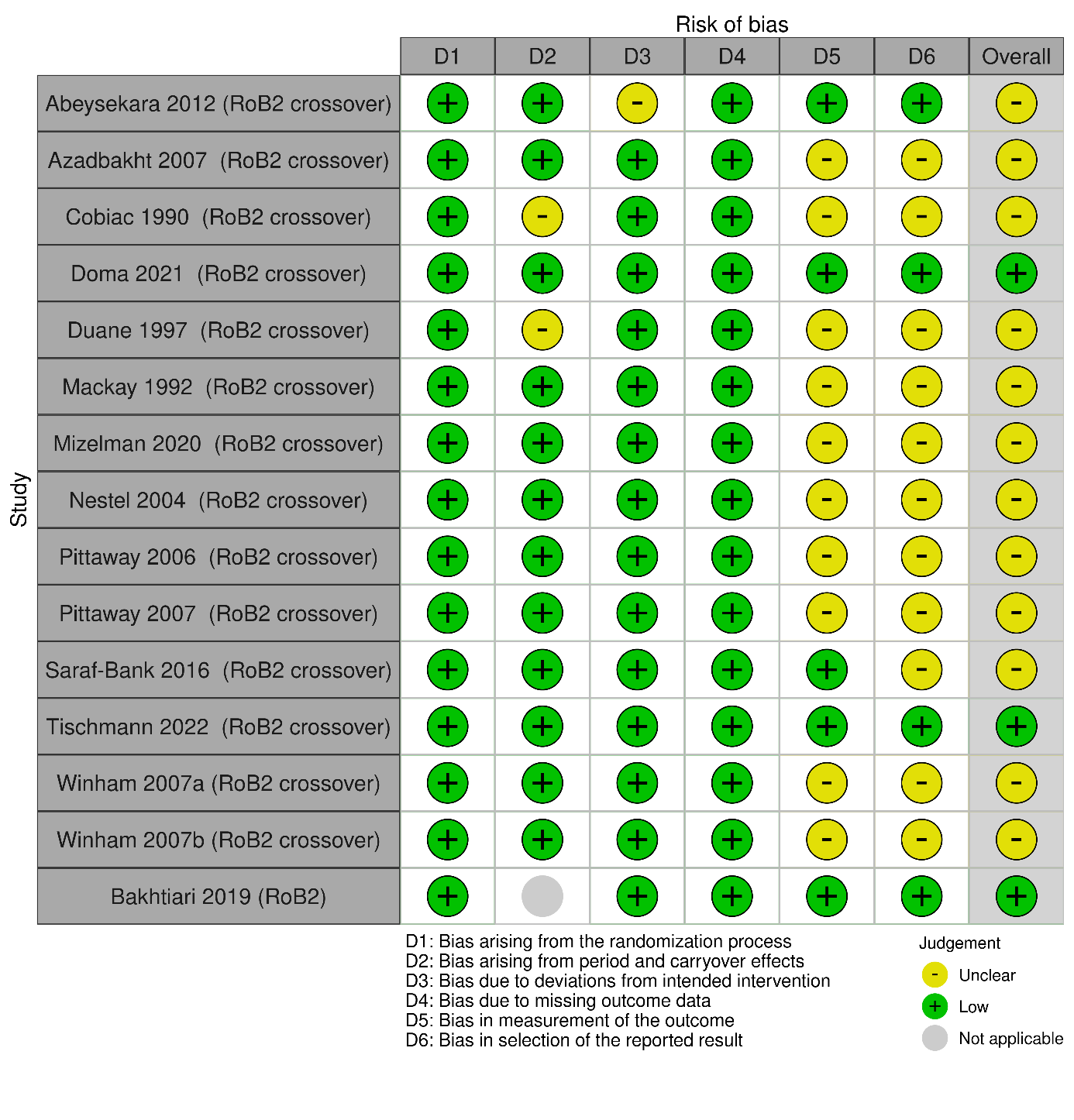
**Supplementary figure 2.** Risk of bias per domain in RCTs (crossover and the single parallel).

**Supplementary figure 3**. Legume consumption and risk of cardiometabolic endpoints in cohort studies, separated by type of exposure. The figures show summary forest plots of pooled relative risk estimates between highest and lowest legume consumption categories and risk of the endpoints: CVD (A), CHD (B), stroke (C), T2D (D).

**A**

**B**


**C**

**D**

**Supplementary figure 4.** Effect of legume intervention vs. control in RCTs, separated by type of intervention. The figures show summary forest plots of pooled mean differences between intervention and control in cardiometabolic risk factors: total cholesterol, mmol/L (A), LDL-cholesterol, mmol/L (B), HDL-cholesterol, mmol/L (C), triglycerides, mmol/L (D), glucose, mmol/L (E), insulin, pmol/L (F), HOMA-IR (G).

**A**

**B**

**C**

**D**

**E**

**F**

**G**

**Supplemental figure 5.** Effect of legume intervention vs. control in intervention studies, separated by risk of bias. The figures show summary forest plots of pooled mean differences between intervention and control in cardiometabolic risk factors: total cholesterol, mmol/L (A), LDL-cholesterol, mmol/L (B), HDL-cholesterol, mmol/L (C), triglycerides, mmol/L (D), glucose, mmol/L (E), insulin, pmol/L (F), HOMA-IR (G).

**A**

**B**

**C**

**D**

**E**

**F**

**Supplementary figure 6**. Contour-enhanced funnel plots for small-study effects estimated with random-effects restricted maximum likelihood models. Red vertical lines correspond to the estimated overall effect size. Total CVD (A), total CHD (B), total stroke (C), type 2 diabetes (D), total choleterol (E), LDL-cholesterol (F), HDL- cholesterol (G), triglycerides (H), insulin (I), glucose (J).

**A**

**B**

**C**

**D**

**E**

**F**

**G**

**H**

**I**

**J**
