# Supplementary material for: Legume consumption in adults and risk of cardiovascular disease and type 2 diabetes: a systematic review and meta-analysis
Source: Food Nutr Res. 2023 May 30;67:10.29219/fnr.v67.9541. doi: 10.29219/fnr.v67.9541 (PMC10243120; doi:10.29219/fnr.v67.9541)
Supplement: Supplementary file 2 [file FNR-67-9541-s002.pdf]

Documentation of literature search

Documentation on the literature search for: Consumption of pulses/legumes and risk of cardiovascular disease and type 2 diabetes and their risk factors: A systematic review

All searches were performed by Hilde Strømme, Academic Librarian, University of Oslo, Library of Medicine and Science on 2 May 2021 and updated by the same person on 16 May 2022. In the update searches publication year was limited to 2021-2022. All results were exported to EndNote and duplicates were removed. For the update search records already identified by the initial search were also removed in EndNote.

Initial search:

**Database**	**Number of retrieved references**
MEDLINE (Ovid)	2793
Embase (Ovid)	5094
Cochrane Central Register of Controlled Trials (Cochrane Library, Wiley)	1723
Scopus	8640
Number of references before deduplication:	18250
Number of references after deduplication:	9951

Update search (limited to publication year 2021-2022):

**Database**	**Number of retrieved references**
MEDLINE (Ovid)	319
Embase (Ovid)	496
Cochrane Central Register of Controlled Trials (Cochrane Library, Wiley)	93
Scopus	919
Number of references before deduplication:	1827
Number of *new* references after deduplication:	820

Initial search 2 May 2021 in Ovid MEDLINE(R) ALL <1946 to April 30, 2021>

1	(diet* or intake or ingest*).mp.	1003979
2	Fabaceae/ or Arachis/ or Cajanus/ or Canavalia/ or Cicer/ or Crotalaria/ or Lens Plant/ or Lupinus/ or Peas/ or Phaseolus/ or Soybeans/ or Vicia/ or Vicia faba/ or Vigna/ or exp Soy Foods/	59460
3	Cardiovascular Diseases/ or Atherosclerosis/ or exp Myocardial Infarction/ or exp Stroke/ or exp Coronary Disease/ or exp Coronary Artery Bypass/ or exp "Diabetes Mellitus, Type 2"/ or exp Insulin Resistance/ or C-Peptide/ or Glucose Intolerance/ or Glycated Hemoglobin A/ or Blood Glucose/ or Hyperglycemia/ or Blood Pressure/ or Hypertension/ or Lipids/ or exp Triglycerides/ or exp Apolipoproteins/ or Cholesterol/ or Cholesterol, HDL/ or Cholesterol, LDL/ or Cholesterol, VLDL/	1615666

UiO : University of Oslo Library
Medical Library

4	1 and 2 and 3	2375
5	(fabaceae or legum* or canavalia or canavalias or crotalaria* or pea or peas or chickpea* or cicer or cicers or garbanzo* or lentil or lentils or lupin* or soy* or tofu or phaseolus or faba or pisum or pisums or pigeonpea or pigeonpeas or bean or beans or kidneybean* or pintobean* or navybean* or cannellinibean* or limabean* or mungbean* or favabean* or blackbean* or drybean* or whitebean* or vicia or (lens adj (plant* or culinar*))).tw,kf.	127579
6	((ACA or anterior cerebral artery or anterior cerebral circulation or anterior choroidal artery or brain or brain stem or brainstem or brain venous or cerebral or heart or heubner* artery or MCA or middle cerebral artery or myocardial or PCA or posterior cerebral artery or posterior choroidal artery or subcortical) adj2 infarct*).tw,kf.	235594
7	((anterior cerebral artery or basilar or benedict or claude or coronary-subclavian steal or dorsolateral medullary or foville or lateral bulbar or lateral medullary or middle cerebral artery or millard-gublar or posterior cerebral artery or posterior inferior cerebellar artery or wallenberg* or weber) adj2 syndrome*).tw,kf.	3502
8	((brain vascular or cerebrovascular) adj2 accident*).tw,kf.	7568
9	((coronary artery or aortocoronary) adj2 bypass*).tw,kf.	45123
10	(coronary adj3 (aneurysm* or arterioscleros#s or artery anastomos#s or disease* or occlusion* or restenos#s or stenos#s or syndrome* or thrombos#s or vasospasm*)).tw,kf.	218357
11	(apoplex* or atherogenesis or atheroscleros#s or cardiogenic shock or heart attack* or middle cerebral artery thrombosis or stroke*).tw,kf.	405575
12	(diabet* adj3 ("2" or "type II" or Adult-Onset or Non Insulin or NonInsulin)).tw,kf.	175557
13	(DM2 or NIDDM or IIDM or MODY or T2DM).tw,kf.	34090
14	(blood adj2 (glucose or sugar*)).tw,kf.	91628
15	(lipid* adj2 (blood or level or profile*)).tw,kf.	54870
16	(blood pressure or cardiometabolic syndrome* or C-peptide or cholesterol or HDL or LDL or VLDL or connecting peptide or diastolic pressure or dysmetabolic syndrome* or glucose intolerance* or HOMA-IR or hyperglycemia* or hypertension or insulin resistance or insulin sensitivity or metabolic syndrome* or metabolic cardiovascular syndrome* or pulse pressure or reaven syndrome X or systolic pressure).tw,kf.	1022347
17	((glycated or glycosylated) adj2 (haemoglobin* or hemoglobin*)).tw,kf.	23726
18	(glycohemoglobin A or Hb A1 or HbA1 or Hb A1a-1 or Hb A1a-2 or Hb A1a+b or Hb A1b or Hb A1c or HbA1c or "hemoglobin A(1)" or hemoglobin A1C).tw,kf.	45773
19	(Apo-B or ApoA or ApoA-II or Apo A-V or Apo A1 or Apo A2 or Apo A5 or APOA5 or Apo AI or ApoB or ApoB48 or ApoC or Apo C or ApoC2 or Apo D or ApoD or ApoE or Apo E or APOE-epsilon* or ApoE2 or Apo E2 or Apo E3 or ApoE3 or Apo E4 or ApoE4 or ApoL or ApoL1 or apolipoprotein* or apoprotein* or enzactin or glycerol trioleate or proapolipoprotein* or triacetin or triacetyl-glycerol* or triacetylglycerol* or triacylglycerol* or trielaidin or triglyceride* or trioleate-glycerin or triolein or trioleoylglycerol or trioleyl glycerol*).tw,kf.	191000
20	1 and 5 and (or/6-19)	5242
21	4 or 20	6029
22	21 not (exp "Animals"/ not (exp "Animals"/ and "Humans"/))	3633
23	22 not (Case Reports or Comment or Congress or Editorial or guideline or Interview or meta analysis or Legal Case or Letter or Meeting Abstract or practice guideline or review or systematic review).pt.	2793

Update search 16 May 2022 Ovid MEDLINE(R) ALL <1946 to May 13, 2022>

1	(diet* or intake or ingest*).mp.	1062486
2	Fabaceae/ or Arachis/ or Cajanus/ or Canavalia/ or Cicer/ or Crotalaria/ or Lens Plant/ or Lupinus/ or Peas/ or Phaseolus/ or Soybeans/ or Vicia/ or Vicia faba/ or Vigna/ or exp Soy Foods/	63269
3	Cardiovascular Diseases/ or Atherosclerosis/ or exp Myocardial Infarction/ or exp Stroke/ or exp Coronary Disease/ or exp Coronary Artery Bypass/ or exp "Diabetes Mellitus, Type 2"/ or exp Insulin Resistance/ or C-Peptide/ or Glucose Intolerance/ or Glycated Hemoglobin A/ or Blood Glucose/ or Hyperglycemia/ or Blood Pressure/ or Hypertension/ or Lipids/ or exp Triglycerides/ or exp Apolipoproteins/ or Cholesterol/ or Cholesterol, HDL/ or Cholesterol, LDL/ or Cholesterol, VLDL/	1710491
4	1 and 2 and 3	2455
5	(fabaceae or legum* or canavalia or canavalias or crotalaria* or pea or peas or chickpea* or cicer or cicers or garbanzo* or lentil or lentils or lupin* or soy* or tofu or phaseolus or faba or pisum or pisums or pigeonpea or pigeonpeas or bean or beans or kidneybean* or pintobean* or navybean* or cannellinibean* or limabean* or mungbean* or favabean* or blackbean* or drybean* or whitebean* or vicia or (lens adj (plant* or culinar*))).tw,kf.	135815
6	((ACA or anterior cerebral artery or anterior cerebral circulation or anterior choroidal artery or brain or brain stem or brainstem or brain venous or cerebral or heart or heubner* artery or MCA or middle cerebral artery or myocardial or PCA or posterior cerebral artery or posterior choroidal artery or subcortical) adj2 infarct*).tw,kf.	248057
7	((anterior cerebral artery or basilar or benedict or claude or coronary-subclavian steal or dorsolateral medullary or foville or lateral bulbar or lateral medullary or middle cerebral artery or millard-gublar or posterior cerebral artery or posterior inferior cerebellar artery or wallenberg* or weber) adj2 syndrome*).tw,kf.	3620
8	((brain vascular or cerebrovascular) adj2 accident*).tw,kf.	8012
9	((coronary artery or aortocoronary) adj2 bypass*).tw,kf.	47027
10	(coronary adj3 (aneurysm* or arterioscleros#s or artery anastomos#s or disease* or occlusion* or restenos#s or stenos#s or syndrome* or thrombos#s or vasospasm*)).tw,kf.	229623
11	(apoplex* or atherogenesis or atheroscleros#s or cardiogenic shock or heart attack* or middle cerebral artery thrombosis or stroke*).tw,kf.	436452
12	(diabet* adj3 ("2" or "type II" or Adult-Onset or Non Insulin or NonInsulin)).tw,kf.	190808
13	(DM2 or NIDDM or IIDM or MODY or T2DM).tw,kf.	38308
14	(blood adj2 (glucose or sugar*)).tw,kf.	98184
15	(lipid* adj2 (blood or level or profile*)).tw,kf.	59079
16	(blood pressure or cardiometabolic syndrome* or C-peptide or cholesterol or HDL or LDL or VLDL or connecting peptide or diastolic pressure or dysmetabolic syndrome* or glucose intolerance* or HOMA-IR or hyperglycemia* or hypertension or insulin resistance or insulin sensitivity or metabolic syndrome* or metabolic cardiovascular syndrome* or pulse pressure or reaven syndrome X or systolic pressure).tw,kf.	1077465
17	((glycated or glycosylated) adj2 (haemoglobin* or hemoglobin*)).tw,kf.	25821
18	(glycohemoglobin A or Hb A1 or HbA1 or Hb A1a-1 or Hb A1a-2 or Hb A1a+b or Hb A1b or Hb A1c or HbA1c or "hemoglobin A(1)" or hemoglobin A1C).tw,kf.	50619

19	(Apo-B or ApoA or ApoA-II or Apo A-V or Apo A1 or Apo A2 or Apo A5 or APOA5 or Apo AI or ApoB or ApoB48 or ApoC or Apo C or ApoC2 or Apo D or ApoD or ApoE or Apo E or APOE-epsilon* or ApoE2 or Apo E2 or Apo E3 or ApoE3 or Apo E4 or ApoE4 or ApoL or ApoL1 or apolipoprotein* or apoprotein* or enzactin or glycerol trioleate or proapolipoprotein* or triacetin or triacetyl-glycerol* or triacetylglycerol* or triacylglycerol* or trielaidin or triglyceride* or trioleate-glycerin or triolein or trioleoylglycerol or trioleyl glycerol*).tw,kf.	201571
20	1 and 5 and (or/6-19)	5595
21	4 or 20	6397
22	21 not (exp "Animals"/ not (exp "Animals"/ and "Humans"/))	3863
23	22 not (Case Reports or Comment or Congress or Editorial or guideline or Interview or meta analysis or Legal Case or Letter or Meeting Abstract or practice guideline or review or systematic review).pt.	2962
24	limit 23 to yr="2021 - 2022"	319

Initial search 2 May 2021 in Embase Classic+Embase <1947 to 2021 April 30>

1	(diet* or intake or ingest*).mp.	1530078
2	Fabaceae/ or Legume/ or Bean/ or mung bean/ or Phaseolus vulgaris/ or Soybean/ or tonka bean/ or velvet bean/ or Vicia faba/ or Chickpea/ or Cowpea/ or Lentil/ or Lupin/ or Lupinus albus/ or Lupinus angustifolius/ or Lupinus luteus/ or Pea/ or Pigeonpea/ or exp soy food/	76316
3	cardiovascular disease/ or atherosclerosis/ or exp heart infarction/ or exp cerebrovascular accident/ or exp coronary artery disease/ or coronary artery bypass graft/ or non insulin dependent diabetes mellitus/ or insulin resistance/ or c peptide/ or glucose intolerance/ or hemoglobin a1c/ or glucose blood level/ or hyperglycemia/ or blood pressure/ or hypertension/ or exp lipid blood level/ or exp apolipoprotein/ or high density lipoprotein cholesterol/ or low density lipoprotein cholesterol/ or very low density lipoprotein cholesterol/	2640434
4	1 and 2 and 3	3334
5	(fabaceae or legum* or canavalia or canavalias or crotalaria* or pea or peas or chickpea* or cicer or cicers or garbanzo* or lentil or lentils or lupin* or soy* or tofu or phaseolus or faba or pisum or pisums or pigeonpea or pigeonpeas or bean or beans or kidneybean* or pintobean* or navybean* or cannellinibean* or limabean* or mungbean* or favabean* or blackbean* or drybean* or whitebean* or vicia or (lens adj (plant* or culinar*))).tw,kw.	143375
6	((ACA or anterior cerebral artery or anterior cerebral circulation or anterior choroidal artery or brain or brain stem or brainstem or brain venous or cerebral or heart or heubner* artery or MCA or middle cerebral artery or myocardial or PCA or posterior cerebral artery or posterior choroidal artery or subcortical) adj2 infarct*).tw,kw.	361257
7	((anterior cerebral artery or basilar or benedict or claude or coronary-subclavian steal or dorsolateral medullary or foville or lateral bulbar or lateral medullary or middle cerebral artery or millard-gublar or posterior cerebral artery or posterior inferior cerebellar artery or wallenberg* or weber) adj2 syndrome*).tw,kw.	5136
8	((brain vascular or cerebrovascular) adj2 accident*).tw,kw.	13614
9	((coronary artery or aortocoronary) adj2 bypass*).tw,kw.	61833
10	(coronary adj3 (aneurysm* or arterioscleros#s or artery anastomos#s or disease* or occlusion* or restenos#s or stenos#s or syndrome* or thrombos#s or vasospasm*)).tw,kw.	341605
11	(apoplex* or atherogenesis or atheroscleros#s or cardiogenic shock or heart attack* or middle cerebral artery thrombosis or stroke*).tw,kw.	652322

UiO : University of Oslo Library
Medical Library

12	(diabet* adj3 ("2" or "type II" or Adult-Onset or Non Insulin or NonInsulin)).tw,kw.	274630
13	(DM2 or NIDDM or IIDM or MODY or T2DM).tw,kw.	56966
14	(blood adj2 (glucose or sugar*)).tw,kw.	149363
15	(lipid* adj2 (blood or level or profile*)).tw,kw.	81276
16	(blood pressure or cardiometabolic syndrome* or C-peptide or cholesterol or HDL or LDL or VLDL or connecting peptide or diastolic pressure or dysmetabolic syndrome* or glucose intolerance* or HOMA-IR or hyperglycemia* or hypertension or insulin resistance or insulin sensitivity or metabolic syndrome* or metabolic cardiovascular syndrome* or pulse pressure or reaven syndrome X or systolic pressure).tw,kw.	1560867
17	((glycated or glycosylated) adj2 (haemoglobin* or hemoglobin*)).tw,kw.	32496
18	(glycohemoglobin A or Hb A1 or HbA1 or Hb A1a-1 or Hb A1a-2 or Hb A1a+b or Hb A1b or Hb A1c or HbA1c or "hemoglobin A(1)" or hemoglobin A1C).tw,kw.	92770
19	(Apo-B or ApoA or ApoA-II or Apo A-V or Apo A1 or Apo A2 or Apo A5 or APOA5 or Apo AI or ApoB or ApoB48 or ApoC or Apo C or ApoC2 or Apo D or ApoD or ApoE or Apo E or APOE-epsilon* or ApoE2 or Apo E2 or Apo E3 or ApoE3 or Apo E4 or ApoE4 or ApoL or ApoL1 or apolipoprotein* or apoprotein* or enzactin or glycerol trioleate or proapolipoprotein* or triacetin or triacetyl-glycerol* or triacetylglycerol* or triacylglycerol* or trielaidin or triglyceride* or trioleate-glycerin or triolein or trioleoylglycerol or trioleyl glycerol*).tw,kw.	272955
20	1 and 5 and (or/6-19)	7261
21	4 or 20	8464
22	21 not ("animal"/ not "human"/)	7640
23	22 not (Conference abstract or Conference paper or Conference review or Editorial or Letter or Note or Short survey or Review).pt.	5094

Update search 16 May 2022 Embase Classic+Embase <1947 to 2022 May 13>

1	(diet* or intake or ingest*).mp.	1591306
2	Fabaceae/ or Legume/ or Bean/ or mung bean/ or Phaseolus vulgaris/ or Soybean/ or tonka bean/ or velvet bean/ or Vicia faba/ or Chickpea/ or Cowpea/ or Lentil/ or Lupin/ or Lupinus albus/ or Lupinus angustifolius/ or Lupinus luteus/ or Pea/ or Pigeonpea/ or exp soy food/	80118
3	cardiovascular disease/ or atherosclerosis/ or exp heart infarction/ or exp cerebrovascular accident/ or exp coronary artery disease/ or coronary artery bypass graft/ or non insulin dependent diabetes mellitus/ or insulin resistance/ or c peptide/ or glucose intolerance/ or hemoglobin a1c/ or glucose blood level/ or hyperglycemia/ or blood pressure/ or hypertension/ or exp lipid blood level/ or exp apolipoprotein/ or high density lipoprotein cholesterol/ or low density lipoprotein cholesterol/ or very low density lipoprotein cholesterol/	2789642
4	1 and 2 and 3	3581
5	(fabaceae or legum* or canavalia or canavalias or crotalaria* or pea or peas or chickpea* or cicer or cicers or garbanzo* or lentil or lentils or lupin* or soy* or tofu or phaseolus or faba or pisum or pisums or pigeonpea or pigeonpeas or bean or beans or kidneybean* or pintobean* or navybean* or cannellinibean* or limabean* or mungbean* or favabean* or blackbean* or drybean* or whitebean* or vicia or (lens adj (plant* or culinar*))).tw,kw.	148763
6	((ACA or anterior cerebral artery or anterior cerebral circulation or anterior choroidal artery or brain or brain stem or brainstem or brain venous or cerebral or heart or heubner* artery	364921

UiO : University of Oslo Library
Medical Library

	or MCA or middle cerebral artery or myocardial or PCA or posterior cerebral artery or posterior choroidal artery or subcortical) adj2 infarct*).tw,kw.	
7	((anterior cerebral artery or basilar or benedict or claude or coronary-subclavian steal or dorsolateral medullary or foville or lateral bulbar or lateral medullary or middle cerebral artery or millard-gublar or posterior cerebral artery or posterior inferior cerebellar artery or wallenberg* or weber) adj2 syndrome*).tw,kw.	4985
8	((brain vascular or cerebrovascular) adj2 accident*).tw,kw.	12712
9	((coronary artery or aortocoronary) adj2 bypass*).tw,kw.	62005
10	(coronary adj3 (aneurysm* or arterioscleros#s or artery anastomos#s or disease* or occlusion* or restenos#s or stenos#s or syndrome* or thrombos#s or vasospasm*)).tw,kw.	338819
11	(apoplex* or atherogenesis or atheroscleros#s or cardiogenic shock or heart attack* or middle cerebral artery thrombosis or stroke*).tw,kw.	690226
12	(diabet* adj3 ("2" or "type II" or Adult-Onset or Non Insulin or NonInsulin)).tw,kw.	287736
13	(DM2 or NIDDM or IIDM or MODY or T2DM).tw,kw.	61976
14	(blood adj2 (glucose or sugar*)).tw,kw.	154726
15	(lipid* adj2 (blood or level or profile*)).tw,kw.	84243
16	(blood pressure or cardiometabolic syndrome* or C-peptide or cholesterol or HDL or LDL or VLDL or connecting peptide or diastolic pressure or dysmetabolic syndrome* or glucose intolerance* or HOMA-IR or hyperglycemia* or hypertension or insulin resistance or insulin sensitivity or metabolic syndrome* or metabolic cardiovascular syndrome* or pulse pressure or reaven syndrome X or systolic pressure).tw,kw.	1615882
17	((glycated or glycosylated) adj2 (haemoglobin* or hemoglobin*)).tw,kw.	33851
18	(glycohemoglobin A or Hb A1 or HbA1 or Hb A1a-1 or Hb A1a-2 or Hb A1a+b or Hb A1b or Hb A1c or HbA1c or "hemoglobin A(1)" or hemoglobin A1C).tw,kw.	100004
19	(Apo-B or ApoA or ApoA-II or Apo A-V or Apo A1 or Apo A2 or Apo A5 or APOA5 or Apo AI or ApoB or ApoB48 or ApoC or Apo C or ApoC2 or Apo D or ApoD or ApoE or Apo E or APOE-epsilon* or ApoE2 or Apo E2 or Apo E3 or ApoE3 or Apo E4 or ApoE4 or ApoL or ApoL1 or apolipoprotein* or apoprotein* or enzactin or glycerol trioleate or proapolipoprotein* or triacetin or triacetyl-glycerol* or triacetylglycerol* or triacylglycerol* or trielaidin or triglyceride* or trioleate-glycerin or triolein or trioleoylglycerol or trioleyl glycerol*).tw,kw.	283238
20	1 and 5 and (or/6-19)	7479
21	4 or 20	8848
22	21 not ("animal"/ not "human"/)	7962
23	22 not (Conference abstract or Conference paper or Conference review or Editorial or Letter or Note or Short survey or Review).pt.	5320
24	limit 23 to yr="2021 - 2022"	496

Initial search 2 May 2021 in Cochrane Central Register of Controlled Trials

#1	(diet* OR intake OR ingest*):ti,ab,kw	129252
#2	([mh ^Fabaceae] OR [mh ^Arachis] OR [mh ^Cajanus] OR [mh ^Canavalia] OR [mh ^Cicer] OR [mh ^Crotalaria] OR [mh ^"Lens Plant"] OR [mh ^Lupinus] OR [mh ^Peas] OR [mh ^Phaseolus] OR [mh ^Soybeans] OR [mh ^Vicia] OR [mh ^"Vicia faba"] OR [mh ^Vigna] OR [mh "Soy Foods"])	1403

#3	(fabaceae OR legum* OR canavalia OR canavalias OR crotalaria* OR pea OR peas OR chickpea* OR cicer OR cicers OR garbanzo* OR lentil OR lentils OR lupin* OR soy* OR tofu OR phaseolus OR faba OR pisum OR pisums OR pigeonpea OR pigeonpeas OR bean OR beans OR kidneybean* OR pintobean* OR navybean* OR cannellinibean* OR limabean* OR mungbean* OR favabean* OR blackbean* OR drybean* OR whitebean* OR vicia OR (lens NEXT (plant* OR culinar*))):ti,ab,kw	6177
#4	[mh ^"Cardiovascular Diseases"]	8415
#5	[mh ^Atherosclerosis]	1396
#6	[mh "Myocardial Infarction"]	11303
#7	[mh Stroke]	10259
#8	[mh "Coronary Disease"]	14030
#9	[mh "Coronary Artery Bypass"]	5482
#10	[mh "Diabetes Mellitus, Type 2"]	18189
#11	[mh "Insulin Resistance"]	6479
#12	[mh ^C-Peptide]	1252
#13	[mh ^"Glucose Intolerance"]	1152
#14	[mh ^"Glycated Hemoglobin A"]	5844
#15	[mh ^"Blood Glucose"]	16312
#16	[mh ^Hyperglycemia]	1960
#17	[mh ^"Blood Pressure"]	26829
#18	[mh ^Hypertension]	17835
#19	[mh ^Lipids]	6435
#20	[mh Triglycerides]	6404
#21	[mh Apolipoproteins]	2025
#22	[mh ^Cholesterol]	5988
#23	[mh ^"Cholesterol, HDL"]	3749
#24	[mh ^"Cholesterol, LDL"]	4794
#25	[mh ^"Cholesterol, VLDL"]	252
#26	((ACA OR "anterior cerebral artery" OR "anterior cerebral circulation" OR "anterior choroidal artery" OR brain OR "brain stem" OR brainstem OR "brain venous" OR cerebral OR heart OR (heubner* NEXT artery) OR MCA OR "middle cerebral artery" OR myocardial OR PCA OR "posterior cerebral artery" OR "posterior choroidal artery" OR subcortical) NEAR/2 infarct*):ti,ab,kw	39794
#27	(("anterior cerebral artery" OR basilar OR benedict OR claude OR "coronary-subclavian steal" OR "dorsolateral medullary" OR foville OR "lateral bulbar" OR "lateral medullary" OR "middle cerebral artery" OR "millard-gublar" OR "posterior cerebral artery" OR "posterior inferior cerebellar artery" OR wallenberg* OR weber) NEAR/2 syndrome*):ti,ab,kw	45
#28	(("brain vascular" OR cerebrovascular) NEAR/2 accident*):ti,ab,kw	13571
#29	(("coronary artery" OR aortocoronary) NEAR/2 bypass*):ti,ab,kw	12211

#30	(coronary NEAR/3 (aneurysm* OR arterioscleros?s OR (artery NEXT anastomos?s) OR disease* OR occlusion* OR restenos?s OR stenos?s OR syndrome* OR thrombos?s OR vasospasm*)):ti,ab,kw	36828
#31	(apoplex* OR atherogenesis OR atheroscleros?s OR cardiogenic shock OR (heart NEXT attack*) OR "middle cerebral artery thrombosis" OR stroke*):ti,ab,kw	68797
#32	(diabet* NEAR/3 ("2" OR "type II" OR "Adult-Onset" OR "Non Insulin" OR NonInsulin)):ti,ab,kw	45737
#33	(DM2 OR NIDDM OR IIDM OR MODY OR T2DM):ti,ab,kw	8036
#34	(blood NEAR/2 (glucose OR sugar*)):ti,ab,kw	41714
#35	(lipid* NEAR/2 (blood OR level OR profile*)):ti,ab,kw	19817
#36	("blood pressure" OR (cardiometabolic NEXT syndrome*) OR "C-peptide" OR cholesterol OR HDL OR LDL OR VLDL OR "connecting peptide" OR "diastolic pressure" OR (dysmetabolic NEXT syndrome*) OR (glucose NEXT intolerance*) OR "HOMA-IR" OR hyperglycemia* OR hypertension OR "insulin resistance" OR "insulin sensitivity" OR (metabolic NEXT syndrome*) OR (metabolic NEXT cardiovascular NEXT syndrome*) OR "pulse pressure" OR "reaven syndrome X" OR "systolic pressure"):ti,ab,kw	177527
#37	((glycated OR glycosylated) NEAR/2 (haemoglobin* OR hemoglobin*)):ti,ab,kw	11106
#38	("glycohemoglobin A" OR "Hb A1" OR HbA1 OR "Hb A1a-1" OR "Hb A1a-2" OR "Hb A1a+b" OR "Hb A1b" OR "Hb A1c" OR HbA1c OR "hemoglobin A(1)" OR "hemoglobin A1C"):ti,ab,kw	22470
#39	("Apo-B" OR ApoA OR "ApoA-II" OR "Apo A-V" OR "Apo A1" OR "Apo A2" OR "Apo A5" OR APOA5 OR "Apo AI" OR ApoB OR ApoB48 OR ApoC OR "Apo C" OR ApoC2 OR "Apo D" OR ApoD OR ApoE OR "Apo E" OR (APOE NEXT epsilon*) OR ApoE2 OR "Apo E2" OR "Apo E3" OR ApoE3 OR "Apo E4" OR ApoE4 OR ApoL OR ApoL1 OR apolipoprotein* OR apoprotein* OR enzactin OR "glycerol trioleate" OR proapolipoprotein* OR triacetin OR (triacetyl NEXT glycerol*) OR triacetylglycerol* OR triacylglycerol* OR trielaidin OR triglyceride* OR "trioleate-glycerin" OR triolein OR trioleoylglycerol OR (trioleyl NEXT glycerol*)):ti,ab,kw	29425
#40	#1 AND (#2 OR #3) AND ({OR #4-#39})	1739
#41	#40 in Trials	1723

Update search 16 May 2022 in Cochrane Central Register of Controlled Trials

#1	(diet* OR intake OR ingest*):ti,ab,kw	136560
#2	([mh ^Fabaceae] OR [mh ^Arachis] OR [mh ^Cajanus] OR [mh ^Canavalia] OR [mh ^Cicer] OR [mh ^Crotalaria] OR [mh ^"Lens Plant"] OR [mh ^Lupinus] OR [mh ^Peas] OR [mh ^Phaseolus] OR [mh ^Soybeans] OR [mh ^Vicia] OR [mh ^"Vicia faba"] OR [mh ^Vigna] OR [mh "Soy Foods"])	1482
#3	(fabaceae OR legum* OR canavalia OR canavalias OR crotalaria* OR pea OR peas OR chickpea* OR cicer OR cicers OR garbanzo* OR lentil OR lentils OR lupin* OR soy* OR tofu OR phaseolus OR faba OR pisum OR pisums OR pigeonpea OR pigeonpeas OR bean OR beans OR kidneybean* OR pintobean* OR navybean* OR cannellinibean* OR limabean* OR mungbean* OR favabean* OR blackbean* OR drybean* OR whitebean* OR vicia OR (lens NEXT (plant* OR culinar*))):ti,ab,kw	6527
#4	[mh ^"Cardiovascular Diseases"]	9013
#5	[mh ^Atherosclerosis]	1492
#6	[mh "Myocardial Infarction"]	11715
#7	[mh Stroke]	11365

#8	[mh "Coronary Disease"]	14594
#9	[mh "Coronary Artery Bypass"]	5622
#10	[mh "Diabetes Mellitus, Type 2"]	19842
#11	[mh "Insulin Resistance"]	7009
#12	[mh ^C-Peptide]	1296
#13	[mh ^"Glucose Intolerance"]	1232
#14	[mh ^"Glycated Hemoglobin A"]	6362
#15	[mh ^"Blood Glucose"]	17368
#16	[mh ^Hyperglycemia]	2048
#17	[mh ^"Blood Pressure"]	27613
#18	[mh ^Hypertension]	18572
#19	[mh ^Lipids]	6686
#20	[mh Triglycerides]	6606
#21	[mh Apolipoproteins]	2081
#22	[mh ^Cholesterol]	6099
#23	[mh ^"Cholesterol, HDL"]	3846
#24	[mh ^"Cholesterol, LDL"]	4941
#25	[mh ^"Cholesterol, VLDL"]	257
#26	((ACA OR "anterior cerebral artery" OR "anterior cerebral circulation" OR "anterior choroidal artery" OR brain OR "brain stem" OR brainstem OR "brain venous" OR cerebral OR heart OR (heubner* NEXT artery) OR MCA OR "middle cerebral artery" OR myocardial OR PCA OR "posterior cerebral artery" OR "posterior choroidal artery" OR subcortical) NEAR/2 infarct*):ti,ab,kw	41565
#27	(("anterior cerebral artery" OR basilar OR benedict OR claude OR "coronary-subclavian steal" OR "dorsolateral medullary" OR foville OR "lateral bulbar" OR "lateral medullary" OR "middle cerebral artery" OR "millard-gublar" OR "posterior cerebral artery" OR "posterior inferior cerebellar artery" OR wallenberg* OR weber) NEAR/2 syndrome*):ti,ab,kw	51
#28	(("brain vascular" OR cerebrovascular) NEAR/2 accident*):ti,ab,kw	14891
#29	(("coronary artery" OR aortocoronary) NEAR/2 bypass*):ti,ab,kw	12516
#30	(coronary NEAR/3 (aneurysm* OR arterioscleros?s OR (artery NEXT anastomos?s) OR disease* OR occlusion* OR restenos?s OR stenos?s OR syndrome* OR thrombos?s OR vasospasm*)):ti,ab,kw	38405
#31	(apoplex* OR atherogenesis OR atheroscleros?s OR cardiogenic shock OR (heart NEXT attack*) OR "middle cerebral artery thrombosis" OR stroke*):ti,ab,kw	73631
#32	(diabet* NEAR/3 ("2" OR "type II" OR "Adult-Onset" OR "Non Insulin" OR NonInsulin)):ti,ab,kw	48667
#33	(DM2 OR NIDDM OR IIDM OR MODY OR T2DM):ti,ab,kw	8640
#34	(blood NEAR/2 (glucose OR sugar*)):ti,ab,kw	44167
#35	(lipid* NEAR/2 (blood OR level OR profile*)):ti,ab,kw	20825
#36	("blood pressure" OR (cardiometabolic NEXT syndrome*) OR "C-peptide" OR cholesterol OR HDL OR LDL OR VLDL OR "connecting peptide" OR "diastolic pressure" OR (dysmetabolic	187467

	NEXT syndrome*) OR (glucose NEXT intolerance*) OR "HOMA-IR" OR hyperglycemia* OR hypertension OR "insulin resistance" OR "insulin sensitivity" OR (metabolic NEXT syndrome*) OR (metabolic NEXT cardiovascular NEXT syndrome*) OR "pulse pressure" OR "reaven syndrome X" OR "systolic pressure"):ti,ab,kw	
#37	((glycated OR glycosylated) NEAR/2 (haemoglobin* OR hemoglobin*)):ti,ab,kw	11814
#38	("glycohemoglobin A" OR "Hb A1" OR HbA1 OR "Hb A1a-1" OR "Hb A1a-2" OR "Hb A1a+b" OR "Hb A1b" OR "Hb A1c" OR HbA1c OR "hemoglobin A(1)" OR "hemoglobin A1C"):ti,ab,kw	23848
#39	("Apo-B" OR ApoA OR "ApoA-II" OR "Apo A-V" OR "Apo A1" OR "Apo A2" OR "Apo A5" OR APOA5 OR "Apo AI" OR ApoB OR ApoB48 OR ApoC OR "Apo C" OR ApoC2 OR "Apo D" OR ApoD OR ApoE OR "Apo E" OR (APOE NEXT epsilon*) OR ApoE2 OR "Apo E2" OR "Apo E3" OR ApoE3 OR "Apo E4" OR ApoE4 OR ApoL OR ApoL1 OR apolipoprotein* OR apoprotein* OR enzactin OR "glycerol trioleate" OR proapolipoprotein* OR triacetin OR (triacetyl NEXT glycerol*) OR triacetylglycerol* OR triacylglycerol* OR trielaidin OR triglyceride* OR "trioleate-glycerin" OR triolein OR trioleoylglycerol OR (trioleyl NEXT glycerol*)):ti,ab,kw	30585
#40	#1 AND (#2 OR #3) AND ({OR #4-#39})	1803
#41	#40 with Cochrane Library publication date Between May 2021 and May 2022, in Trials	93

Intial search 2 May 2021 in Scopus

The same search was re-run on 16 May 2022 and restricted to publication year 2021-2022

TITLE-ABS-KEY (("diet*" OR "intake" OR "ingest*") AND ("fabaceae" OR "legum*" OR "canavalia" OR "canavalias" OR "crotalaria*" OR "pea" OR "peas" OR "chickpea*" OR "cicer" OR "cicers" OR "garbanzo*" OR "lentil" OR "lentils" OR "lupin*" OR "soy*" OR "tofu" OR "phaseolus" OR "faba" OR "pisum" OR "pisums" OR "pigeonpea" OR "pigeonpeas" OR "bean" OR "beans" OR "kidneybean*" OR "pintobean*" OR "navybean*" OR "cannellinibean*" OR "limabean*" OR "mungbean*" OR "favabean*" OR "blackbean*" OR "drybean*" OR "whitebean*" OR "vicia" OR ("lens" W/0 ("plant*" OR "culinar*"))) AND ((("ACA" OR "anterior cerebral artery" OR "anterior cerebral circulation" OR "anterior choroidal artery" OR "brain" OR "brain stem" OR "brainstem" OR "brain venous" OR "cerebral" OR "heart" OR "heubner* artery" OR "MCA" OR "middle cerebral artery" OR "myocardial" OR "PCA" OR "posterior cerebral artery" OR "posterior choroidal artery" OR "subcortical") W/1 "infarct*") OR (("anterior cerebral artery" OR "basilar" OR "benedict" OR "claude" OR "coronary-subclavian steal" OR "dorsolateral medullary" OR "foville" OR "lateral bulbar" OR "lateral medullary" OR "middle cerebral artery" OR "millard-gublar" OR "posterior cerebral artery" OR "posterior inferior cerebellar artery" OR "wallenberg*" OR "weber") W/1 "syndrome*") OR (("coronary artery" OR "aortocoronary") W/1 "bypass*") OR ("coronary" W/2 ("aneurysm*" OR "arterioscleros*" OR "artery anastomos")) OR ("apoplex*" OR "atherogenesis" OR "atheroscleros*" OR "cardiogenic shock" OR "heart attack*" OR "middle cerebral artery thrombosis" OR "stroke*") OR ("diabet*" W/2 ("2" OR "type II" OR "Adult-Onset" OR "Non Insulin" OR "NonInsulin")) OR ("DM2" OR "NIDDM" OR "IIDM" OR "MODY" OR "T2DM") OR ("blood" W/1 ("glucose" OR "sugar*")) OR ("lipid*" W/1 ("blood" OR "level" OR "profile*")) OR ("blood pressure" OR "cardiometabolic syndrome*" OR "C-peptide" OR "cholesterol" OR "HDL" OR "LDL" OR "VLDL" OR "connecting peptide" OR "diastolic pressure" OR "dysmetabolic syndrome*" OR "glucose intolerance*" OR "HOMA-IR" OR "hyperglycemia*" OR "hypertension" OR "insulin resistance" OR "insulin sensitivity" OR "metabolic syndrome*" OR "metabolic cardiovascular syndrome*" OR "pulse pressure" OR "reaven syndrome X" OR "systolic pressure") OR (("glycated" OR "glycosylated") W/1 ("haemoglobin*" OR "hemoglobin*")) OR ("glycohemoglobin A" OR "Hb A1" OR "HbA1" OR "Hb A1a-1" OR "Hb A1a-2" OR "Hb A1a+b" OR "Hb A1b" OR "Hb A1c" OR "HbA1c" OR "hemoglobin A(1)" OR "hemoglobin A1C") OR ("Apo-B" OR "ApoA" OR "ApoA-II" OR "Apo A-V" OR "Apo A1" OR "Apo A2" OR "Apo A5" OR "APOA5" OR "Apo AI" OR "ApoB" OR "ApoB48" OR "ApoC" OR "Apo C" OR "ApoC2" OR "Apo D" OR "ApoD" OR "ApoE" OR "Apo E" OR "APOE epsilon*" OR "ApoE2" OR "Apo E2" OR "Apo E3" OR "ApoE3" OR "Apo E4" OR "ApoE4" OR "ApoL" OR "ApoL1" OR "apolipoprotein*" OR "apoprotein*" OR "enzactin" OR "glycerol trioleate" OR "proapolipoprotein*" OR "triacetin" OR "triacetyl glycerol*" OR

"triacetylglycerol*" OR "triacylglycerol*" OR "trielaidin" OR "triglyceride*" OR "trioleate-glycerin" OR "triolein" OR "trioleoylglycerol" OR "trioleyl glycerol*"))) AND (EXCLUDE (DOCTYPE, "re") OR EXCLUDE (DOCTYPE, "cp") OR EXCLUDE (DOCTYPE, "ch") OR EXCLUDE (DOCTYPE, "ed") OR EXCLUDE (DOCTYPE, "le") OR EXCLUDE (DOCTYPE, "sh") OR EXCLUDE (DOCTYPE, "bk") OR EXCLUDE (DOCTYPE, "cr"))
